# CENP-A chromatin disassembly in stressed and senescent murine cells

**DOI:** 10.1038/srep42520

**Published:** 2017-02-10

**Authors:** Sabrine Hédouin, Giacomo Grillo, Ivana Ivkovic, Guillaume Velasco, Claire Francastel

**Affiliations:** 1 Université Paris Diderot, Sorbonne Paris Cité, Epigenetics and Cell Fate, CNRS UMR7216, 75205 Paris cedex, France

## Abstract

Centromeres are chromosomal domains essential for genomic stability. We report here the remarkable transcriptional and epigenetic perturbations at murine centromeres in genotoxic stress conditions. A strong and selective transcriptional activation of centromeric repeats is detected within hours. This is followed by disorganization of centromeres with striking delocalization of nucleosomal CENP-A, the key determinant of centromere identity and function, in a mechanism requiring active transcription of centromeric repeats, the DNA Damage Response (DDR) effector ATM and chromatin remodelers/histone chaperones. In the absence of p53 checkpoint, activated transcription of centromeric repeats and CENP-A delocalization do not occur and cells accumulate micronuclei indicative of genomic instability. In addition, activated transcription and loss of centromeres identity are features of permanently arrested senescent cells with persistent DDR activation. Together, these findings bring out cooperation between DDR effectors and loss of centromere integrity as a safeguard mechanism to prevent genomic instability in context of persistent DNA damage signalling.

Centromeres are specialized chromosomal regions that serve as an assembly platform for attachment of kinetochore and mitotic spindle, which are essential for faithful segregation of genetic material during cell division^1^. Hence, maintenance of centromere identity and function is tightly linked to maintenance of genome stability and integrity.

Most centromeres assemble on repeated sequences, yet no sequence conservation between species helps to genetically define their position. However, a centromere-specific variant of histone H3, CENP-A, serves as a key epigenetic determinant of centromere identity and kinetochore assembly through the generation of a unique chromatin organization^2^,^3^. Furthermore, centromeric transcripts are emerging as integral components of centromeric chromatin, participating in CENP-A deposition on chromatin and centromere function^4^,^5^,^6^,^7^,^8^,^9^,^10^. Their levels are tightly regulated during cell cycle^7^ and their unscheduled accumulation has been observed in human diseases^11^,^12^,^13^ and stress conditions^14^,^15^,^16^. Recently, we functionally linked this accumulation to perturbed centromere architecture and function leading to genome instability and aneuploidy in the mouse^5^. Hence, accumulation of centromeric transcripts is probably not a mere consequence of a physiopathological state and might represent a conserved feature of the cellular stress response.

Agents and processes that inflict damage to DNA and cause genotoxic stress are particularly deleterious since they severely compromise genome integrity. To counteract the adverse effects of DNA damage and their transmission to daughter cells, cells have developed sophisticated and coordinated surveillance mechanisms^17^,^18^. The multifactorial DNA damage response (DDR) is the central regulator of this network. It senses the DNA lesion and transmits the damage signal through the activation of signalling cascades to initiate DNA repair and stall damaged cells until DNA lesions are repaired. DDR is orchestrated by the ATM and ATR kinases, which phosphorylate a multitude of proteins to modulate cellular response depending on the type of damage, cellular context and intensity and duration of stress^19^. The appropriate response is triggered by effector pathways allowing DNA repair, cell cycle arrest, senescence, apoptosis or cell death, among which the p53 pathway is probably the main effector downstream of DNA strand breaks and activation of ATM/ATR^20^.

Here, we aimed at establishing the kinetics of epigenetic and transcriptional perturbations that impact centromere identity in response to stress. We report that murine centromeric transcripts accumulate upon DNA damage within a few hours, in a manner that is dependent on the DDR effector p53. This is followed by disorganization of centromeric chromatin associated with the striking relocation of parental nucleosomal CENP-A, in a manner that also requires ATM-mediated signalling pathway and chromatin chaperones/remodelling factors, the most prominent being the FACT (facilitates chromatin transcription) complex. We found that perturbations to transcription and centromeric architecture are also hallmarks of senescent cells where the DDR is activated independently of the presence of exogenous genotoxic stressors^21^. As a whole, our data uncovered a novel crosstalk between DDR effectors and dynamics at centromeric chromatin, where a p53/ATM-dependent disruption of centromeric structure and identity may trigger safeguard mechanisms to prevent genomic instability in cases of persistent DNA damage signalling.

## Results

### Accumulation of DNA damage leads to CENP-A mislocalization

**Figure 1:**
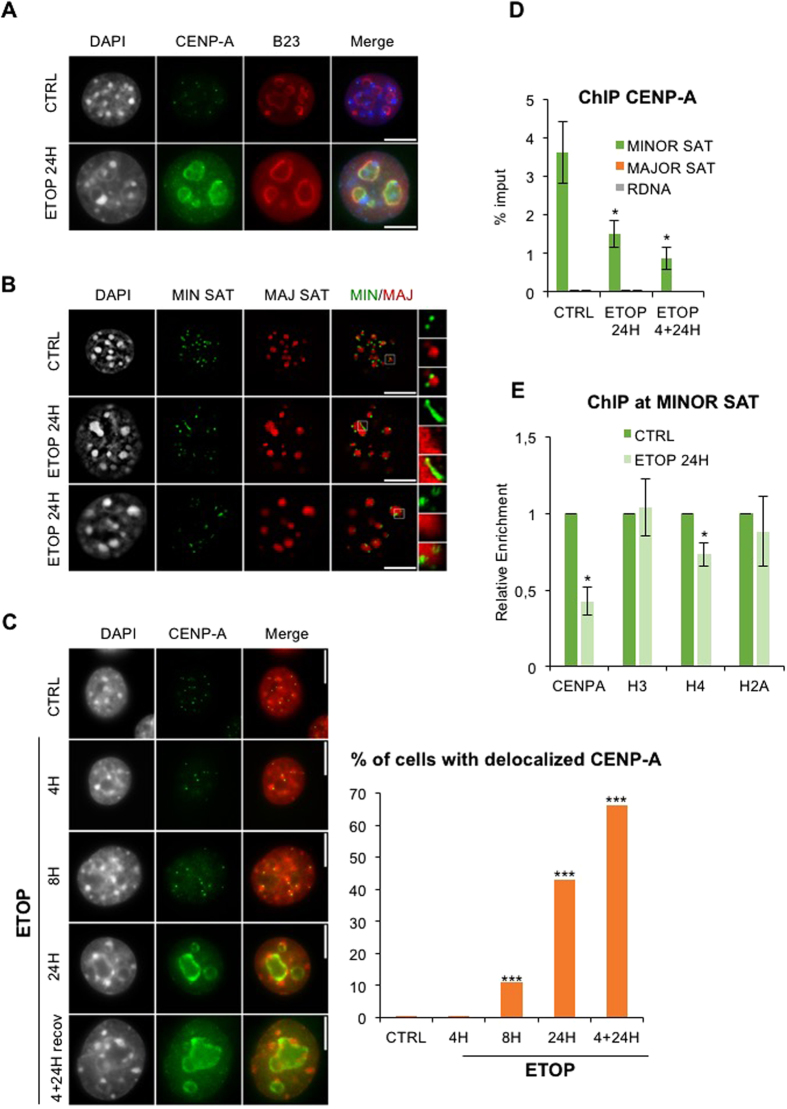
CENP-A is delocalized from centromeric repeats in response to genotoxic stress. (**A**) CENP-A localization in NIH/3T3 cells treated with 10 μM ETOP for 24 hr, analyzed by IF using anti-murine CENP-A and secondary anti-rabbit antibody conjugated to Alexa-488 (green). Antibodies against B23 (nucleophosmin) and anti-mouse antibody conjugated to Alexa-594 (red) were used to mark nucleoli. DAPI was used to stain nuclei (blue). (**B**) Architecture of minor and major satellites repeats in NIH/3T3 cells treated with 10 μM ETOP for 24 hr, analyzed by DNA-FISH with specific probes fluorescently labelled with Alexa 488- (green) or Cy3- (red) conjugated dUTP, respectively. Insets are magnified an additional 3X. (**C**) CENP-A localization analyzed in kinetics experiments on NIH/3T3 cells treated with 10 μM ETOP for 4, 8, 24 hr, or 4 hr followed by a 24 hr recovery period, using immunofluorescence as in (**A**). Percentage of cells that displayed delocalized CENP-A exemplified in (**A**), bottom panel, are indicated on the histogram on the right (n > 200 for each condition). P-values (Chi-square test) comparing control and ETOP-treated cells: ***p < 0.001. (**D**) CENP-A enrichment at minor satellite, major satellite and rDNA repeats after ETOP treatment, analyzed by ChIP-qPCR. Data are normalized to the input. (**E**) CENP-A and canonical histones enrichment at minor satellite repeats after ETOP treatment, analyzed by ChIP-qPCR. Data are normalized to the input then to the control condition (CTRL) arbitrarily set at 1. Error bars represent S.E.M of at least three independent experiments. P-values (unpaired t-test) comparing controls (CTRL) and drug treatments: *p < 0.05 (unpaired t-test). CTRL: untreated cells. Images represent one focal plan. Scale bar, 10 μm.

We treated murine NIH/3T3 cells with a representative panel of genotoxic agents under conditions known to promote various types of DNA damage (Table S1) as revealed by accumulation of phosphorylated histone variant H2A.X (γH2A.X) and stabilization of p53 (Figure S1A). We monitored the impact of various drug treatments on cell cycle by FACS (Figure S1B). Centromere architecture was assessed in single cells using immunofluorescence (IF) to follow CENP-A localization and DNA-FISH using probes specific for centromeric repeats termed minor satellites in the mouse. In untreated cells, CENP-A staining and minor satellite repeats adopted the typical punctate pattern in the vicinity of chromocenters^22^, composed of pericentromeric major satellite repeats or visualized as dense DAPI staining (Fig. 1A and B; top rows). We first focused on Etoposide (ETOP), a potent inducer of DNA double strand breaks (DSB), as a paradigm for studying the impact of DNA damage on centromeres. We found that CENP-A became remarkably mislocalized away from its normal location and occupied the periphery and inside of the nucleolus marked by B23/nucleophosmin signal (Fig. 1A; bottom row). Likewise, ETOP treatment led to the disorganization of centromeric DNA, mainly characterized by stretched rather than punctiform minor satellite signals, while chromocenters remained globally less affected (Fig. 1B; two bottom rows).

To exclude an effect restricted to topoisomerase II inhibition by ETOP, we tested other genotoxic drugs that have various impacts on DNA (Table S1) and on cell cycle (Figure S1B). We found that Zeocin (ZEO), Mitomycin-C (MMC) and, to a lesser extent, Hydroxyurea (HU) in the conditions used, led to CENP-A relocation away from centromeric foci (Figure S2A). In contrast, forced cell-cycle arrest in G2/M after Nocodazole (NOC) treatment, a poison of microtubules, was not sufficient to promote CENP-A delocalization (Figure S2A).

Kinetics experiments following genotoxic stress showed that, while maintaining the typical punctate pattern nearby chromocenters during the first 4 hr of ETOP treatment, CENP-A became visibly mislocalized by 8 hr, and further occupied the nucleoplasm after 24 hr (Fig. 1C). In contrast, other centromeric proteins like CENP-B and CENP-C mainly displayed the typical centromeric punctate pattern, despite being slightly enriched in the nucleoplasm (Figure S2B and C). Although CENP-A delocalization seemed to be a relatively early event in the response to DNA damage, we followed the appearance of cleaved Caspase-3, one of the earliest molecular events at the onset of apoptosis. We did not detect cleaved Caspase-3 before 24 hr of ETOP treatment (Figure S2D). The signal was also undetectable in cells treated for 4 hr that have recovered from stress after a 24 hr period (Figure S2D). Hence, CENP-A relocation does not seem to be a direct consequence of the activation of pro-apoptotic signals. Rather, it may precede cellular changes.

Etoposide forms non-repairable DSB^23^ while cells progressively accumulate in G2/M (Figure S1B). Interestingly, whereas a 4 hr ETOP treatment did not promote visible CENP-A delocalization (Fig. 1C), a 24 hr recovery period (4 + 24H) during which DNA damage signalling and γH2A.X signal persisted (Figure S2D) and most cells arrested in G2/M (Figure S1B) promoted CENP-A delocalization in more than 60% of the cells (Fig. 1C; lower row). Similar data were obtained with 4 hr ZEO or MMC treatments followed by a 24 hr recovery period (Figure S3A), time point at which γH2A.X is still detectable (Figure S3B). These data support a link between persistence of DNA damage and centromeres loss of identity through CENP-A delocalization. To confirm this hypothesis, we treated cells with lower doses of ETOP (2 μM instead of 10 μM) to allow cells to repair DNA damage, as shown by γH2A.X that returned to baseline levels after a 24 hr recovery from the 4 hr ETOP treatment (Figure S3B). In addition, cells were only transiently arrested in the cell cycle when treated with lower doses of ETOP. Indeed, in contrast to treatment with 10 μM ETOP that led to sustained cell cycle arrest, treatment with 2 μM ETOP allowed cells to resume cell cycle after the recovery period as shown by the increased number of cells in G1 after a 24 hr recovery from the 4 hr treatment (Figure S1B). Under these conditions, the percentage of cells with delocalized CENP-A decreased from nearly 70% to less than 40%, suggesting that CENP-A reoccupies its normal location when DNA damage is repaired (Figure S3A), although we cannot exclude that undamaged cells with or normal CENP-A location take over in the culture during the recovery period.

To confirm the observations made by microscopy, we used Chromatin Immunoprecipitation (ChIP) that showed a decreased CENP-A occupancy at centromeric repeats after ETOP treatment relative to untreated control cells, with a 2.4 fold after 24 hr and almost 4 fold after 4 hr ETOP+ 24 hr recovery (Fig. 1D) that parallels the percentage of cells with delocalized CENP-A (Fig. 1C). In contrast to CENP-A, centromere content in canonical histone H3 did not vary significantly following genotoxic stress (Fig. 1E), whereas content in histone H4 showed a reproducible 1.5-fold reduction after 24 hr ETOP treatment. ChIP experiments also showed that CENP-A did not accumulate at ectopic sites in the nearby pericentromeric major satellite repeats, nor in ribosomal DNA repeats (rDNA) despite its accumulation close to nucleoli (Fig. 1D).

### DNA damage leads to nucleosomal CENP-A delocalization

**Figure 2:**
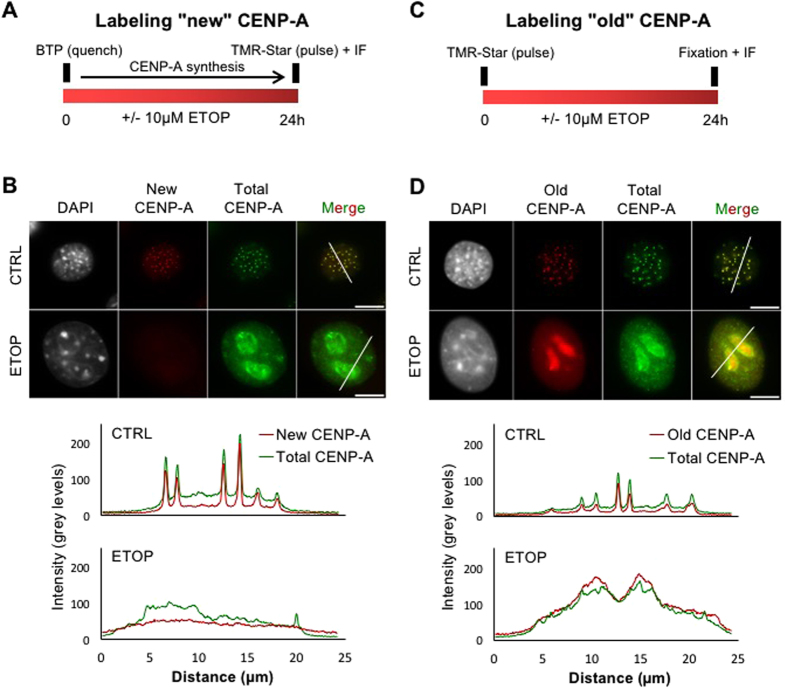
Parental CENP-A is delocalized from centromeres upon DNA damage. (**A**) Schematic representation of the assay used to monitor sub-nuclear localization of newly synthesized CENP-A-SNAP proteins after ETOP treatment using fluorescence microscopy on NIH/3T3 cells stably expressing SNAP-tagged CENP-A. Pre-existing SNAP-tagged CENP-A was quenched with a non-fluorescent substrate (BTP) so that only histones neo-synthetized during the chase period can be further labelled with tetramethylrhodamine (TMR-Star; red) during the pulse step. ETOP treatment was performed during the chase and cells were fixed after 24 hr followed by an IF as in Fig. 1A. (**B**) Detection of total CENP-A using anti-CENP-A antibodies (green) and tagged parental CENP-A (red) following protocol A. Fluorescence intensity line scan through each nucleus (white bar) are represented below images and show the broad nucleolar profiles of total (green line) CENP-A in response to ETOP whereas newly synthesized exogenous CENP-A (SNAP; red line), normally loaded in G1, cannot be incorporated nor accumulates in G2-arrested cells. (**C**) Schematic representation of the assay used to monitor sub-nuclear localization of pre-existing CENP-A proteins after ETOP treatment, using fluorescence microscopy on NIH/3T3 cells stably expressing SNAP-tagged CENP-A. Pre-existing CENP-A-SNAP proteins were labelled with TMR-Star followed by a chase in presence or absence of ETOP. Cells were fixed after 24 hr and IF was performed as in Fig. 1A. (**D**) Same as in (**B**) except that cells were processed following protocol C. Fluorescence intensity line scans through each nucleus show regular punctate pattern in control cells for both exogenous parental (SNAP; red line) and total (green line) CENP-A, whereas ETOP-treated cells show broader signals.

The dynamics of parental nucleosomal and newly synthesized CENP-A is tightly regulated during cell cycle^24^,^25^,^26^,^27^. We took advantage of the SNAP-tag technology that allows distinguishing the fate of parental or newly synthesized histones^28^. We generated NIH/3T3 cell lines expressing the fused CENP-A-SNAP protein, and used quench-chase-pulse or pulse-chase imaging protocols (see the Methods section). Quenching of parental SNAP histones followed by a 24 hr chase of the non-fluorescent substrate in the presence or absence of ETOP, before labelling of newly synthesized CENP-A with the fluorescent substrate TMR-Star (Fig. 2A), showed that ectopic CENP-A was correctly incorporated in centromeric chromatin of untreated cells (Fig. 2B). In contrast, and consistent with cells arresting in G2 while CENP-A is incorporated in G1, newly synthesized CENP-A was not deposited in centromeres nor close to the nucleolus of ETOP-treated cells (Fig. 2B). Fluorescent labelling of the SNAP-tagged CENP-A (Fig. 2C) before a 24 hr ETOP treatment showed that parental nucleosomal CENP-A histones were mostly relocated close to the nucleolus concomitantly with endogenous CENP-A, as shown by line scan analysis (Fig. 2D, bottom panels). These experiments suggested that CENP-A dispersion in response to DNA damage results from eviction of parental nucleosomal histones.

### ATM is required for CENP-A delocalization following DNA damage

**Figure 3:**
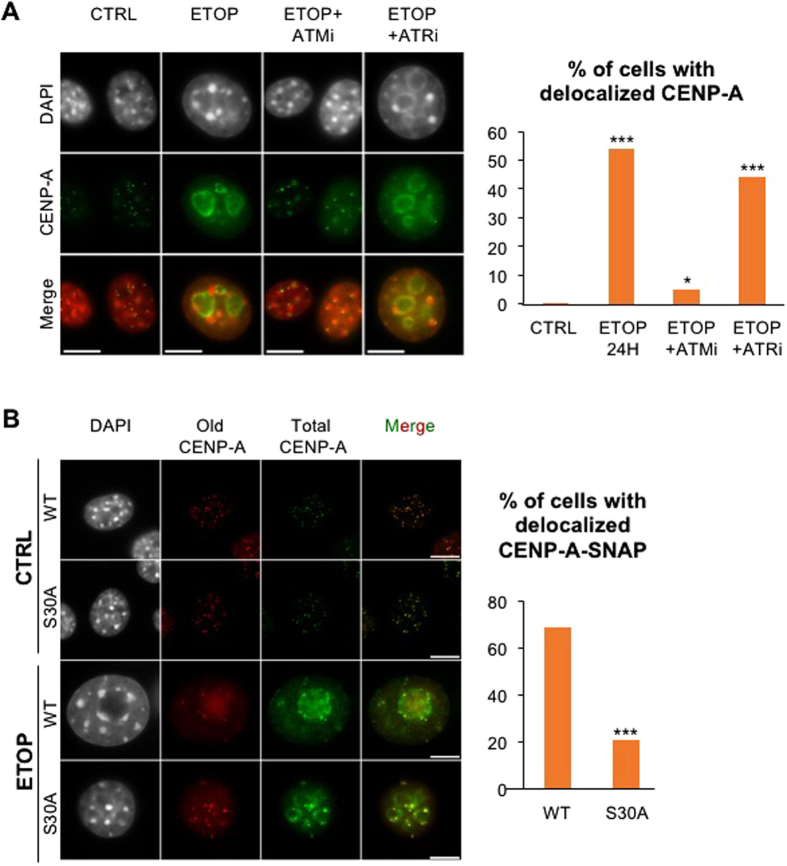
ATM signaling and a phosphorylatable S30 residue are required for CENP-A delocalization. (**A**) CENP-A localization in NIH/3T3 cells co-treated with ETOP and specific inhibitors of ATM (ATMi) or ATR (ATRi) for 24 hr, analyzed as in Fig. 1A. Percentage of cells that displayed CENP-A delocalization is indicated on the histogram on the right (n > 300 cells). (**B**) Sub-nuclear localization of pre-existing CENP-A-SNAP proteins, either WT or S30A mutant, after a 24 hr ETOP treatment was analyzed as in Fig. 2. Images represent one focal plan. Scale bar, 10 μm. P-values (Chi-square test) comparing controls (CTRL) and drug treatments: ***p < 0.001.

CENP-A mislocalization was observed in genotoxic stress conditions. Thus, we assessed the contribution of ATM and ATR kinase signalling cascades that play central roles in cellular response to DNA damage^19^. We found that inhibition of ATM (ATMi), but not that of ATR (ATRi), prevented stress-mediated CENP-A delocalization (Fig. 3A). While DNA damage persists in the presence of genotoxic stressor, its signalling is impaired by ATMi. Interestingly, the reduced CENP-A delocalization observed with ATMi but not ATRi correlated with override of the G2/M cell-cycle block (Figure S3C).

We noticed the presence of a phosphorylation consensus site for ATM (SQ motif) at serine 30 of murine CENP-A. Mutation of this residue into a non-phosphorylatable alanine (S30A) did not prevent deposition of CENP-A-SNAP suggested by its presence at centromeric foci (Fig. 3B). In contrast, delocalization of the parental CENP-A-SNAP S30A mutant version following ETOP treatment was clearly reduced compared to that of the CENP-A-SNAP WT protein (Fig. 3B). Delocalization of total CENP-A upon DNA damage was equivalent between CENP-A-SNAP WT and S30A cell lines (50 and 60%, respectively) and comparable to that of endogenous CENP-A in untransfected cells (Fig. 1C). However, within the population of cells with delocalized total CENP-A, the percentage of cells with delocalized SNAP-tagged CENP-A dropped from 70% for the WT to 20% for the S30A mutant (Fig. 3B), suggesting that both the ATM signalling pathway and a phosphorylatable Ser30 are required for CENP-A eviction in response to DNA damage in association with cell-cycle arrest.

### Activated transcription of centromeric repeats precedes CENP-A dispersal

CENP-A delocalization was detectable only after 8 hr of genotoxic stress (Fig. 1C). Therefore, we assessed the accumulation of centromeric transcripts, another feature of stressed cells reported in various stress conditions (Reviewed in refs 29,30). Various genotoxic conditions led to a strong increase in levels of centromeric transcripts as measured by RT-qPCR, relative to untreated cells (Figure S4A). This is in contrast to non-genotoxic stressors like heat shock (HS) or ethanol (EtOH) that had only modest effects in conditions commonly used (Figure S4A), suggesting that increased transcription of centromeric repeats requires a context of DNA damage as evidenced by accumulation of γ-H2A.X and accumulation of the cyclin-dependent kinase inhibitor 1A p21Cip/CDKN1A (p21^Cip^) (Figure S4A).

**Figure 4:**
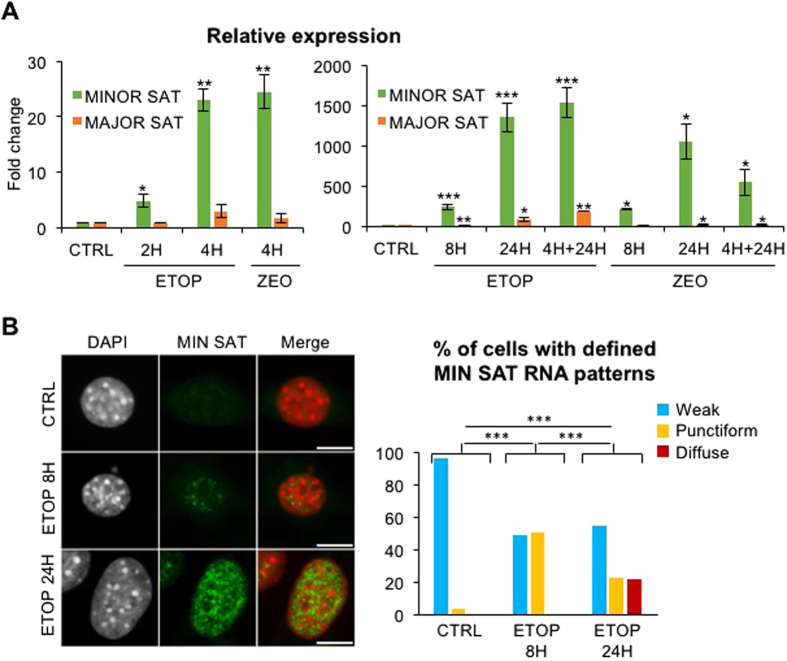
Transcriptional activation of minor satellite repeats in response to genotoxic stress. (**A**) Activated transcription of minor and major satellites in response to ETOP or ZEO treatments, analyzed by RT-qPCR. Short time-point series are shown on the left panel, and longer time-point series or a 4 hr treatment followed by a 24 hr recovery from stress (4 h + 24 h) are shown on the right panel. P-values (unpaired t-test) comparing controls (CTRL) and drug treatments: ***p < 0.001; **p < 0.01; *p < 0.05. CTRL: untreated cells. (**B**) Subnuclear localization of centromeric transcripts in single cells after 8 or 24 hr ETOP treatment, analyzed by RNA-FISH as in Fig. 1B. Histogram shows percentage of cells presenting weak (like in CTRL lane), punctiform (like in ETOP 8 h lane) or diffuse (like in ETOP 24 h lane) minor satellite RNA patterns from RNA-FISH images shown on the left (n > 200 cells counted in each conditions). Error bars represent S.E.M of at least three independent experiments. Images represent one focal plan. Scale bar, 10 μm. P-values comparing the repartition in the three categories at different time points of ETOP treatment: ***p < 0.001 (chi-square test).

Kinetic analysis in response to ETOP or ZEO revealed that levels of centromeric transcripts increased within a few hours, with a 5-fold increase by 2 hr of ETOP (Fig. 4A; left panel) reaching levels up to a thousand-fold in 24 hr (Fig. 4A; right panel). Tandemly repeated pericentromeric major satellite repeats were also induced although with a slower kinetics, with a 2 to 3-fold increase by 4 hr with either drug (Fig. 4A; left panel), and reaching apparent lower levels compared to minor satellites transcription, with a 100-fold increase at 24 hr (Fig. 4A; right panel). In contrast, telomeric or rDNA tandem repeats, interspersed repeats like long (LINE) or short interspersed nuclear elements (SINE) and transposable elements like intracisternal A-particle (IAP), did not show changes in their transcripts levels upon DNA damage (Figure S4B), suggesting the selective activation of centromeric repeats in response to genotoxic stress.

We performed RNA-FISH to assess accumulation of centromeric transcripts at the single cell level. In agreement with previous reports^5^,^7^, centromeric transcripts were hardly detectable in control cycling cells (Fig. 4B). In contrast, centromeric transcripts first accumulated into discrete dots typical of centromeric foci detected in the vicinity of chromocenters in almost half of the cell population after 8 hr of ETOP treatment, suggesting that they remain associated with their transcription sites. Spreading of the signal throughout the nucleoplasm appeared in 20% of the cells after 24 hr of treatment (Fig. 4B), raising the interesting possibility that the impact of increased levels of centromeric transcripts may not be restricted to local perturbations and may affect other nuclear compartments and functions.

Accumulation of murine minor satellites is regulated during cell cycle with a peak in G2/M phase^7^. However, levels reached in G2-arrested ETOP- or ZEO-treated cells were always 10-fold higher than in NOC-arrested cells (Figure S4A). Moreover, high levels of centromeric transcripts were also observed in cells treated with HU and MMC (Figure S4A) that accumulated in G1 or in S (Figure S1B). Hence, accumulation of centromeric transcripts is not a mere consequence of a cell-cycle arrest in G2 but rather the result of an increased transcription or stabilization of these transcripts in response to genotoxic insults. Indeed, inhibition of the transcriptional machinery using the RNA polymerase II (RNA Pol II) inhibitor Actinomycin D (ACTD) together with ETOP treatment abolished the observed strong accumulation of minor satellite transcripts (Figure S5A) as well as CENP-A delocalization (Figure S5B), suggesting that genotoxic stress promotes transcriptional activation of centromeric repeats and that this activated transcription is required for CENP-A relocation. However, forced expression of minor satellite repeats carried out as before^5^ and leading, 24 hr post-transfection, to levels similar to that obtained with a 24 hr ETOP treatment, (Figure S5C) was not sufficient to affect CENP-A endogenous localization (Figure S5D).

### A p53 WT context is required for activated transcription

One of the main effects of DNA damage is the activation of ATM/ATR signaling^17^ and rapid stabilization of the transcription factor p53^20^, which in turn activates downstream effectors for an appropriate cellular response to DNA damage. We found that neither ATMi nor ATRi affected levels of centromeric transcripts compared to ETOP treated cells (Figure S6A), suggesting that transcription of centromeric transcripts is activated in DNA damage conditions but independently of ATM/ATR signalling. It also confirmed that high levels of centromeric repeats are not sufficient to promote eviction of CENP-A since ATMi prevented CENP-A delocalization (Fig. 3A) but not accumulation of satellite repeat transcripts (Figure S6A).

**Figure 5:**
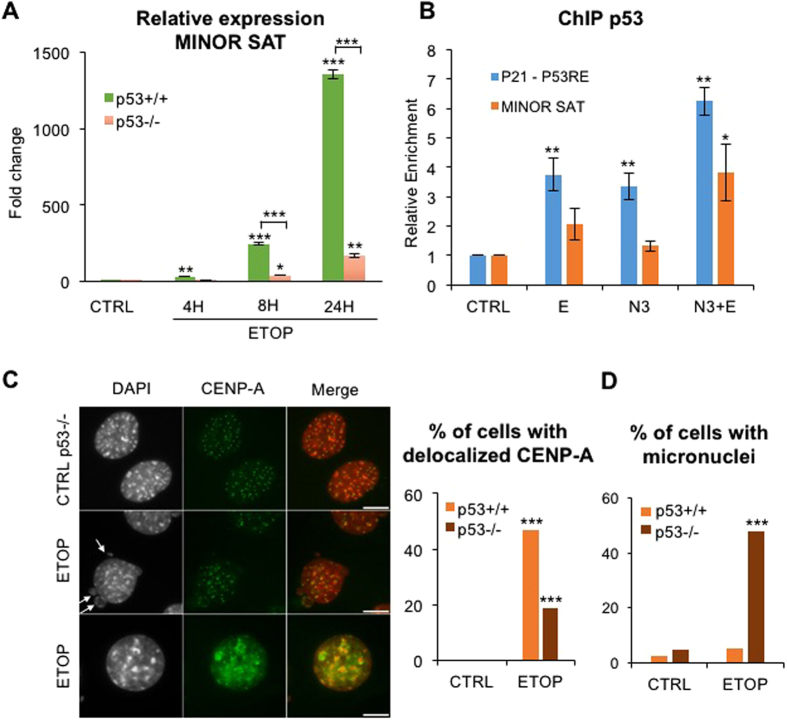
Transcription of centromeric repeats in response to genotoxic stress requires a wildtype p53 context. (**A**) Transcription of minor satellite repeats in WT (NIH3T3; p53^+/+^) or null (p53^−/−^) p53 contexts following ETOP treatment at the indicated time points, was analyzed as in Fig. 4A. *p < 0.05, **p < 0.01, ***p < 0.001 (unpaired t-test). (**B**) Enrichment of endogenous p53 at the p53 responsive element found at p21^CIP^ promoter (p21-p53RE) or at minor satellite repeats in NIH/3T3 cells after a 4 hr treatment with ETOP, Nutlin-3 (N3) or both (N3 + E), analyzed by ChIP-qPCR. Data are normalized to the input then to the control condition. *p < 0.05, **p < 0.01 (unpaired t-test). (**C**) CENP-A localization in p53^−/−^ fibroblasts treated with ETOP for 24 hr, analyzed as in Fig. 1A. Percentage of cells with delocalized CENP-A in p53^−/−^ cells compared to p53^+/+^ cells are represented in the adjacent graph (n > 300 cells). Arrows point to the presence of micronuclei. ***p < 0.001 (chi-square test). (**D**) Percentage of cells with micronuclei in p53^−/−^ cells compared to p53^+/+^ cells (n > 300 cells). ***p < 0.001 (chi-square test). CTRL: untreated cells; E: ETOP; N3: Nutlin-3a. Error bars represent S.E.M of at least three independent experiments. Images represent one focal plan. Scale bar, 10 μm.

We then subjected p53-null murine embryonic fibroblasts (MEF) to the two different doses of ETOP used previously, which led to increased levels of γH2A.X regardless the p53 context, indicating that ATM/ATR signalling is intact in these cells. We verified that the levels of p53 were undetectable (Figure S6B), and that transcription and protein levels of its downstream target p21^Cip^ were activated at both doses in NIH/3T3 cells with wildtype-p53 (WT-p53) but not in p53 null cells (p53^−/−^) (Figure S6B). We found that the levels of minor satellite transcripts did not increase in the absence of p53 after 4 hr of ETOP treatment (Fig. 5A and S6B) and 14-times less compared to WT-p53 cells at later time points (Fig. 5A), suggesting that minor satellite transcription was not delayed but rather severely impaired in a p53-null cellular context.

In non-genotoxic conditions of p53 stabilization, using the small molecule inhibitor of MDM2/p53 interaction Nutlin-3a, transcription from minor satellite repeats was not enhanced, although a slight effect was observed on p53-direct target gene *CDKN1A* transcription (Figure S6C). In contrast, stabilization of p53 by Nutlin-3 potentiated ETOP-mediated transcriptional activation of minor satellite repeats by 3-fold (Figure S6C). ChIP assays showed that, in ETOP-treated cells, p53 had a substantial ability to bind to a consensus single binding site found in minor satellite repeats, although to a lesser extent than to the strong double binding sites described in *CDNK1A* promoter (Fig. 5B). This binding increased by 2-fold in cells treated by both ETOP and Nutlin-3 in correlation with the observed increased transcription (Figs 5B and S6C). These results suggest that stabilized p53 may bind to non-canonical sites in minor satellite repeats, although this binding and the consequent activated transcription of centromeric repeats appears to require the DDR signalling.

CENP-A maintained its default localization in 80% of p53-null cells (Fig. 5C), consistent with both increased transcription of minor satellite transcripts and genotoxic stress signalling being required for CENP-A delocalization. Interestingly, while maintaining the normal CENP-A localization, cells with a defective p53 checkpoint showed an increased micronuclei formation (Figs 5C, arrows; and Fig. 5D) and accumulated in the culture with more than a 2n genomic content (Figure S6D) indicative of defective mitosis and genomic instability.

### Histone chaperone FACT is required for DNA damage-induced CENP-A dispersal

**Figure 6:**
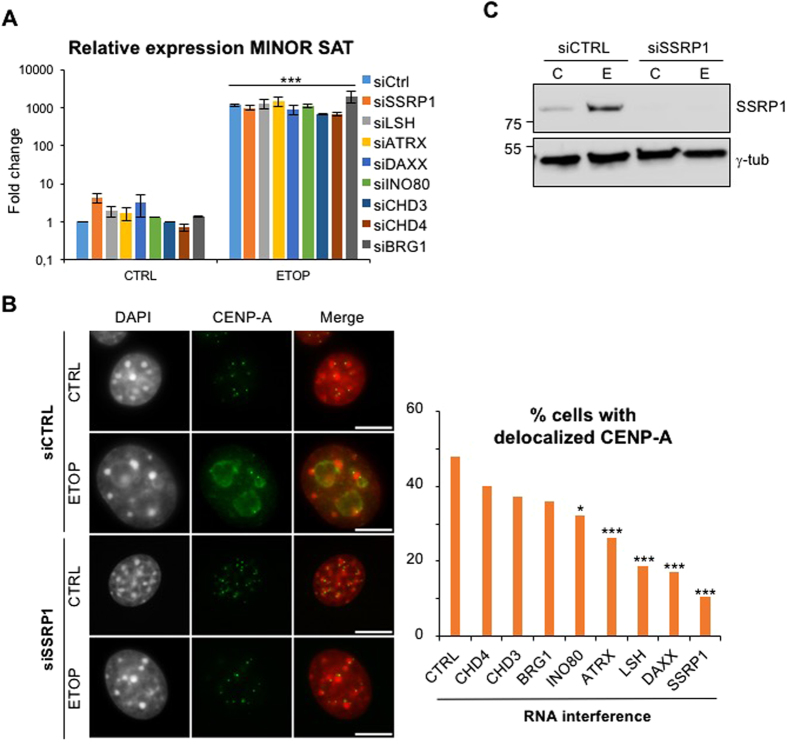
CENP-A delocalization is dependent on chromatin remodelers and the FACT complex. (**A**) Minor satellites transcription following knock down of SSRP1 or other chromatin remodelers/chaperones, was analyzed by qRT-PCR as in Fig. 4A. Histogram represents relative fold change values normalized to U6 RNA levels. Value in untreated cells (CTRL) transfected with control siRNA was arbitrarily set at one. Error bars represent S.E.M of at least 3 independent experiments. ***p < 0.001 (unpaired t-test). (**B**) Impact of SSRP1 knock down, compared to control siRNAs, on CENP-A delocalization in NIH/3T3 cells after a 24 hr ETOP treatment, analyzed as in Fig. 1A. Percentage of cells that displayed CENP-A delocalization in ETOP-treated cells after knock down of SSRP1 or other chromatin remodelers/chaperones are indicated on the histogram on the right (n > 300 cells). Images represent one focal plan. Scale bar, 10 μm. ***p < 0.001 (chi-square test). (**C**) Efficiency of SSRP1 protein knock down using specific compared to control siRNAs, analyzed by western blot. Levels of γ-tubulin served as a loading control.

Since activated centromeric transcription in stress conditions, but not centromeric transcripts themselves, was required for CENP-A delocalization, we further tested whether it could act through chromatin remodelling. We used siRNA-mediated knockdown of a panel of chromatin remodelers for which a number of studies have shown their role at centromeric and pericentromeric repeats (Table S2 and references included). We also focused on the FACT complex, an ATP-independent histone chaperone first discovered as promoting transcriptional elongation^31^ through nucleosome destabilization^32^. In addition, FACT was shown to co-purify with CENP-A nucleosomes^33^, and its subunit SSRP1 to be required for centromeric localization of CENP-A^34^. We found that the levels of centromeric transcripts in ETOP-treated cells were unaffected by reduced levels of the chromatin remodelers/chaperones tested (Fig. 6A). In conditions in which we were able to obtain more than 50% reduction in transcripts levels (Figure S7) the percentage of cells with mislocalized CENP-A after ETOP treatment was largely decreased depending on the remodelling/chaperone factor tested (Fig. 6B). The most striking result was obtained following knockdown of SSRP1 expression, confirmed by a strong decrease in the levels of both mRNA and protein (Fig. 6C), which resulted in less than 10% of cells with delocalized CENP-A after a 24 hr ETOP treatment compared to almost 50% in cells transfected with control siRNAs (Fig. 6B). As a whole, these data revealed cooperation between activated transcription and chromatin chaperones/remodelers to relocate CENP-A in DNA damage conditions.

### CENP-A dispersal is also a feature of permanently arrested senescent cells

Genotoxic conditions leading to CENP-A delocalization and activated transcription of minor satellite repeats led to transcriptional activation of p21^Cip^, indicative of a pre-senescent state (Figure S4A). In primary murine embryonic fibroblasts (MEF), accumulation of minor satellite transcripts also correlated with CENP-A delocalization, although with apparent different kinetics and lower magnitude that may reflect differences of cellular context and the increased sensitivity of primary cells to genotoxic stress. Indeed, a statistically significant and reproducible 2-fold increased transcription of centromeric transcripts was detectable after 4 hr of ETOP treatment together with relocation of CENP-A. These events correlated with increased expression of the senescent marker β-galactosidase (β-gal) and of cell cycle inhibitors like Cyclin-Dependent Kinase Inhibitor 2 A Ink4/p16^INK4a^ (p16^INK4a^) that started being detectable after 24 hr ETOP treatment (Figure S8A,B). We thus assessed centromere integrity in a context of homogeneous populations of senescent cells, where cells are permanently arrested independently of a genotoxic stressor, while maintaining high levels of ATM signalling and stabilized p53^21^.

**Figure 7:**
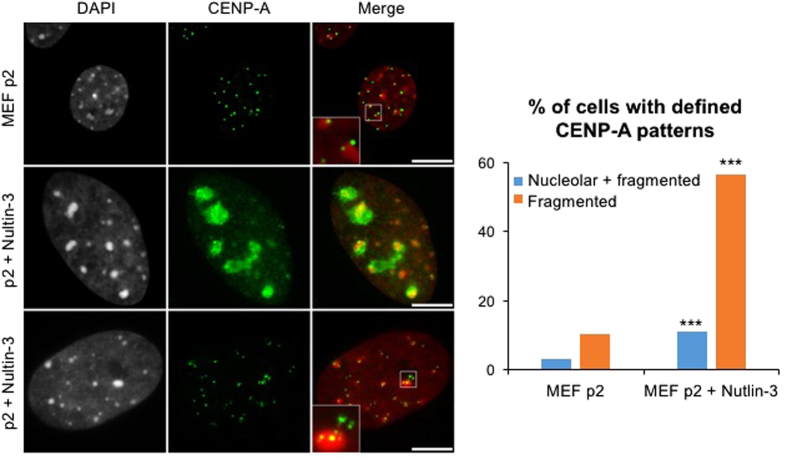
Perturbation of centromeres in senescent cells. CENP-A localization in Nutlin-3-induced senescent cells, analyzed as in Fig. 1A. Percentage of cells showing CENP-A nucleolar (as in middle panel) or fragmented (as in lower panel) patterns are represented on the histogram (n > 200 cells). Images represent one focal plan. Scale bar, 10 μm. ***p < 0.001 (chi-square test).

We first used MEFs as a classical model system as they rapidly lose their proliferation potential after a few passages in culture. MEFs at passage 6 (p6) stained positive for senescence-associated endogenous β-gal in more than 80% of the cells (Figure S8C), and exhibited higher levels of p21^Cip^, p16^INK4a^ and stabilization of p53 (Figure S8D). We also induced senescence in primary cells through the known Nutlin-3a-forced stabilization of p53^35^ that showed the same features as above (Figure S8E). Levels of centromeric transcripts in senescent MEFs-p6 or Nutlin-treated MEFs were 16 and 5-fold higher compared to the same cells at passage 2 (p2), respectively (Figure S9A,B). CENP-A was mislocalized in both cases of senescent cells (Figs 7 and S9C). Compared to punctiform foci observed in control cells at p2, CENP-A formed stretched and rather fragmented signals in 40% of the MEFs-p6 cells (Figure S9C, middle panel). An additional 20% of the cells showed both stretched signals and partial accumulation close to the nucleolus (Figure S9C, lower panel). This effect was even more pronounced following Nutlin-3-forced p53 stabilization with almost 60% of the cells showing aberrant CENP-A localization (Fig. 7). DNA-FISH showed that centromeric architecture was drastically perturbed since, in addition to the known perturbation of pericentromeric heterochromatin foci detected by dense DAPI staining or major satellite probes, centromeric minor satellite signals were also highly disorganized and stretched in senescent cells (Figure S9D).

## Discussion

Our data revealed that murine centromeres are strikingly disassembled in genotoxic stress conditions in a manner that is dependent on the main DDR effectors. Centromeric repeats are selectively and rapidly transcriptionally activated in a p53-dependant but ATM-independent manner. Transcription of centromeric repeats has been suggested to play structural role in centromere or kinetochore integrity in many species (Reviewed in ref. 29. However, we report here that, in combination with activated DDR, it leads to and is required for the drastic structural disassembly of centromeric chromatin in the form of ATM and chromatin remodelers/chaperones-dependent delocalization of its epigenetic mark CENP-A. Remarkably, these features are also hallmarks of permanently arrested senescent cells where the DDR is activated, consistent with the role of p53 and ATM in maintenance of the senescent state and suggestive of a causal link. Together, these data shed light on molecular mechanisms through which activated transcription of murine centromeric repeats may operate as an effector in conditions of sustained DNA damage signalling to trigger safeguard mechanisms and prevent cells from dividing to preserve genomic integrity.

In unstressed conditions, the transcription of murine centromeric transcripts is tightly regulated during the cell cycle^7^. Here, we showed that transcription of centromeric repeats is rapidly activated in response to genotoxic stress, within hours. Interestingly, this response seems to be selective for centromeric repeats, at least in the first hours of the response, since other repetitive elements, either in tandem or interspersed, were not activated. This is in contrast with studies on heat or osmotic shocks that revealed transcriptional activation of human pericentromeric satellite type III repeats, but not that of centromeric alpha satellites^14^,^15^,^16^,^36^. Although satellite repeats transcription seems to be a conserved feature of the stress response, and in absence of sequence conservation among species, the question of any selectivity depending on the type of stress or species is unclear. However, it is interesting to note that conditions where DDR activation is persistent, in cases of non repairable damage or in senescent MEF cells, are characterized by high levels of centromeric transcripts and sustained cell cycle arrest. In addition, a WT-p53 content, known to trigger stable cell-cycle arrest after DNA damage^37^, is also required for transcriptional activation of minor satellite transcripts. Hence, it is tempting to speculate that it is the function of centromeres that is targeted under persistent stress signalling where stable cell cycle arrest is required whereas mild conditions that allow cells to recover from heat shock for instance preserve this function.

We showed that persistent stress signalling and senescence are also characterized by the striking dispersion of CENP-A away from its default localization at centromeric repeats. In fact, perturbed architecture of centromeric repeats seems to be a conserved feature of senescent cells in bovine^38^, human^39^ and murine cells (this study), and most likely relies on CENP-A depletion at centromeres. Indeed, reduced levels of CENP-A levels have been previously reported in human senescent cells, and their forced down regulation by RNA interference leads to premature senescence^40^. We reported here that, in the absence of p53, centromeric transcriptional activation and delocalization of CENP-A do not occur, while cells accumulate micronuclei. Therefore, CENP-A down regulation or relocation away from centromeres might act as a defence mechanism to maintain genomic stability and cell viability by preventing centromere-defective cells from undergoing cell division. Altogether, these data functionally link activated transcription and chromatin remodelling at centromeric regions with stable proliferative halt characteristic of senescent cells or cells subjected to persistent DNA damage, by altering centromere identity and function and hence, preventing cell division.

Transcriptional activation of centromeric repeats in genotoxic stress conditions was detected before CENP-A delocalization suggesting that it may promote or participate in chromatin remodelling process and CENP-A delocalization from centromeric regions at later time points. In support of this hypothesis, we found that ectopically expressed minor satellite transcripts were not sufficient to induce CENP-A relocation. In addition, knock down of several chromatin remodelers and chaperones prevented CENP-A relocation without affecting centromeric repeats transcription. Among these factors, knock down of FACT complex subunit SSRP1 showed the most dramatic effects. The FACT complex emerged as a versatile factor in the control of chromatin dynamics at centromeres depending on cellular contexts, promoting CENP-A deposition during mitosis^34^,^41^ or preventing pervasive deposition^42^,^43^,^44^. Other chromatin remodelers like LSH/HELLS, ATRX and DAXX, for which a role in centromere remodelling has also been reported^36^,^45^,^46^, may cooperate in the eviction process since their loss of function also prevented CENP-A eviction, although to a lesser extent than that of SSRP1. Interestingly, this seems to be independent of the known role of FACT on transcription of centromeric repeats^31^ since loss of function of these factors did not prevent their transcription, suggesting that other factors are implicated.

Chromatin is highly dynamic in response to DNA damage, including post-translational modification of histones and deposition of histone variants at sites of DNA damage as integral parts of the DDR to coordinate efficient signalling and repair^47^. The DDR kinases are involved in genome surveillance and in sensing DNA damage^19^. When activated in the presence of genome threatening insults, they rapidly phosphorylate several hundreds of target proteins^48^. As mentioned above, phosphorylation of histones, like the histone variant H2A.X, participates in chromatin dynamics at strand breaks and is one of the earliest events in the DDR^49^. We further showed that the DDR kinase ATM, but not ATR, was required to promote CENP-A delocalization in response to DNA damage caused by a panel of genotoxic agents. We showed that the rather late DDR-mediated relocation of CENP-A required the ATM kinase and a phosphorylatable consensus SQ motif at Ser30. Under these conditions, it is difficult to assess whether CENP-A is one of the hundreds of targets of ATM signalling pathway, but our data support the view that CENP-A delocalization is mediated by sustained ATM signalling in contexts of persistent DNA damage or senescent cells.

Given that CENP-A nucleosomes may represent only a small fraction of total centromeric nucleosomes in human cells^50^, the slight but reproducible decrease in histone H4 content but not that of canonical H3 suggests that DNA damage and activated centromeric transcription trigger active eviction of a subset of CENP-A nucleosomes from centromeric repeats. This is remarkable since CENP-A nucleosomes are very stable once incorporated into centromeric sequences^51^,^52^. CENP-A deposition and maintenance at the centromere is orchestrated by numerous factors and so far, CENP-A depletion from centromeres has only been linked to perturbations of its deposition machinery^8^,^26^,^27^,^53^. Interestingly, active eviction and degradation of CENP-A has been shown in the case of its ectopic incorporation in yeast and fly chromosome arms^43^,^51^,^54^,^55^. However, in stressed murine cells, we did not find evidence for ectopic incorporation of CENP-A in other DNA repeats although the protein showed apparent accumulation nearby nucleoli. Then, why delocalized CENP-A is not degraded remains a puzzling question.

CENP-A was shown to be very rapidly and transiently recruited at sites of laser induced DNA damage in human and mouse cells^56^, or in drosophila cells depleted for the histone fold protein Chrac14 after MMS treatment^57^. However, it is worth noting that the CENP-A relocation that we observed in response to DNA damage is a rather late event and therefore may not participate in DNA repair *per se* but rather, may be a consequence of prolonged stress and persistent DDR as in senescent cells, promoting or involved in maintenance of cell cycle arrest.

The functional intricacy between DDR and chromatin remodelling at specialized chromosomal domains has been long known in the context of telomeres^58^,^59^. Remarkably, non-canonical p53-binding sites in subtelomeric or centromeric regions confer enhancer-like functions for transcriptional activation at telomeric repeat-containing RNA (TERRA)^60^ or centromeric repeats (this study), required to elicit chromatin changes that will further prevent the cell from dividing. Despite these similarities, in humans, proliferative arrest and replicative senescence are directly linked to telomeres length and function^58^,^61^. In contrast, murine cells enter senescence while maintaining long telomeres. In addition, murine CENP-A presents a consensus site for ATM phosphorylation. Our study uncovered an original link between DDR effectors and centromeres whereby centromere activated transcription and loss of identity is a prominent feature of cell cycle-arrested stressed and senescent murine cells. Hence, whether organisms evolved compensatory mechanisms to efficiently delay or halt the cell cycle to safeguard their genome remains a puzzling question.

## Experimental Procedures

Full experimental procedures are provided in the Supplemental Information section.

### Cell lines and transfections

Mouse embryonic fibroblasts (MEFs), NIH/3T3 cells and their CENP-A-SNAP derivatives were cultured and transfected as described^62^. CENP-A–SNAP was constructed by inserting a PCR-generated murine CENP-A cDNA into pSNAPf vector (New England Biolabs) in frame with the SNAP tag. Mutation of the Ser-30 in alanine (S30A) was generated using the Q5^®^ Site-Directed Mutagenesis Kit according to the manufacturer’s recommendations (New England Biolabs). The eukaryotic expression vector containing four minor satellite repeats units under the control of the CMV promoter was generated as described previously^5^.

### SNAP quench and pulse labelling

We followed previously described protocols for SNAP quench-chase-pulse or pulse-chase imaging^63^. SNAP-tagged histones were pulse-labelled with 2 μM SNAP-cell TMR-star (New England Biolabs) for 15 min or quenched with 2 μM SNAP-cell Block (BTP, New England Biolabs) for 30 min. After quenching or pulse labelling, cells were washed twice with PBS and then incubated in complete medium for 30 min to allow excess compound to diffuse from cells. Then, cells were washed twice with PBS and incubated for 24 h in complete medium containing or not 10 μM Etoposide. Labelling of total CENP-A was then monitored by immunofluorescence as described below.

### Immunofluorescence

Immunofluorescence was performed as previously described^5^.

### DNA & RNA FISH

Cells were directly grown on Superfrost Plus microscope slides (Menzel–Glaser, Braunschweig, Germany) then fixed and permeabilized as described above. DNA-FISH and RNA-FISH were performed as previously described^5^.

### Microscopy

Image acquisition was performed at room temperature on a fluorescence microscope (Axioplan 2; Zeiss) with a Plan-Neofluar 100X/1.3 NA oil immersion objective (Zeiss) using a digital cooled camera (CoolSNAP fx; Photometrics) and METAMORPH 7.04 software (Roper Scientific, Trenton, NJ). Images presented correspond to one focal plane. Analysis was performed by scoring at least 150 cells in each experiment.

### siRNA and plasmid transfection

siRNA purchased from Sigma or Eurofins MWG Operon (Table S5) were transfected into cells using Interferin (Polyplus transfection) following manufacturer’s instructions.

### RNA extraction and analysis of gene expression

Total RNA from cell lines was isolated using TRI Reagent^®^ (Sigma) according to manufacturer’s instructions. Contaminant genomic DNA was eliminated with TURBO DNA-free kit (Ambion). Real-time PCR was performed using the LightCycler^®^ DNA Master SYBR Green I mix (Roche) supplemented with 0.2 μM specific primer pairs (Table S6) and analysed by the comparative CT(ΔΔCT) method using U6 RNA as an invariant RNA. Each data shown in RT-qPCR analysis is the result of at least three independent experiments performed on at least three independent RNA extractions.

### Nuclear extracts and Western blot

Nuclear fractionation and western blot analysis was performed as before^62^.

### Chromatin Immunoprecipitation

ChIP was essentially performed as described^62^. Sequences of primers are listed in Table S6.

## Additional Information

**How to cite this article**: Hédouin, S. *et al*. CENP-A chromatin disassembly in stressed and senescent murine cells. *Sci. Rep.* **7**, 42520; doi: 10.1038/srep42520 (2017).

**Publisher's note:** Springer Nature remains neutral with regard to jurisdictional claims in published maps and institutional affiliations.

## Supplementary Material

Supplementary Information and Figures
