# Supplementary material for: CENP-A chromatin disassembly in stressed and senescent murine cells
Source: Sci Rep. 2017 Feb 10;7:42520. doi: 10.1038/srep42520 (PMC5301216; doi:10.1038/srep42520)

CENP-A chromatin disassembly in stressed and senescent murine cells

Sabrine Hédouin, Giacomo Grillo, Ivana Ivkovic, Guillaume Velasco and Claire Francastel*

Université Paris Diderot, Sorbonne Paris Cité, Epigenetics and Cell Fate, CNRS UMR7216, 75205 Paris cedex, France

* Correspondance to:
Claire Francastel
UMR7216 - Epigénétique et Destin Cellulaire
Université Paris 7 Diderot - Bâtiment Lamarck - 4ème étage
Case Courrier 7042
35, rue Hélène Brion
75205 Paris Cedex 13
e-mail: claire.francastel@univ-paris-diderot.fr

FULL EXPERIMENTAL PROCEDURES

Cell lines and transfections

Mouse embryonic fibroblasts (MEFs) isolated from 12.5 days post conception (d.p.c.) embryos, NIH/3T3 cells and their CENP-A-SNAP derivatives were cultured in complete media (DMEM supplemented with 10% fetal bovine serum, 100 U/mL penicillin and 100μg/mL streptomycin, all from Life Technologies). CENP-A–SNAP was constructed by inserting a PCR-generated murine CENP-A cDNA flanked by NheI and EcoRI sites into the corresponding sites of pSNAPf vector (New England Biolabs) in frame with the SNAP tag. Mutation of the Ser-30 in alanine (S30A) was generated using the Q5® Site-Directed Mutagenesis Kit according to the manufacturer's recommendations (New England Biolabs) using the CENP-A-SNAP plasmid as a template. NIH/3T3 clones expressing CENP-A-SNAP WT and S30A were generated by stable transfection of plasmids using jetPEI (Polyplus transfection) and selection by G418 (1mg/ml; Gibco). Clones were expanded and examined by immunofluorescence and by Western blot using an anti-SNAP antibody (New England Biolabs) to identify lines expressing the CENP-A–SNAP fusion protein at levels smaller or comparable to that of the endogenous CENP-A, exhibiting the typical punctate pattern in the vicinity of chromocenters interphase cells.

The eukaryotic expression vector containing four minor satellite repeat units under the control of the CMV promoter in a pcDNA3 vector was generated as described previously (Bouzinba-Segard et al., 2006). As a control vector, we used the same vector in which the minor satellite sequences were replaced by an irrelevant sequence of the same size. Transient transfections of NIH/3T3 cells were performed using jetPEI (Polyplus transfection) according to manufacturer's recommendations, in conditions allowing a transfection efficiency of 60% as assessed by transfection with a pEGFP-N1 vector (Clontech).

Drug treatments

Cells were treated with the following conditions unless stated otherwise: 10μM Etoposide, 1mM Hydroxyurea, 10μg/ml Mitomycin C, 10μM Nocodazole, 5μg/ml Actinomycin D, 1% EtOH (all from Sigma), 200μg/ml Zeocin (Invitrogen), 50μM ATM inhibitor (KU-55933, Selleckchem) or 10μM ATR inhibitor (VE-821, Selleckchem). Duration of treatments is

indicated in figure legends. For global UVC exposure, adherent cells were washed in PBS and exposed to 20J/m² UVC (254 nm) using a low-pressure mercury lamp, then incubated in complete media and harvested 24h later. Conditions were set using a VLX-3W dosimeter (Vilbert-Lourmat). For heat shock, adherent cells were washed in PBS, overlaid with complete medium pre-warmed at 42°C and incubated at 42°C for 1 h. After heat shock, the medium was replaced with pre-warmed medium at 37°C. Cells were allowed to recover from that point on for 1 hr before harvesting for further experiments.

SNAP quench and pulse labelling

SNAP-tagged histones were pulse-labelled with 2 µM SNAP-cell TMR-star (New England Biolabs) for 15 min or quenched with 2 µM SNAP-cell Block (BTP, New England Biolabs) for 30 min. After quenching or pulse labelling, cells were washed twice with PBS and then incubated in complete medium for 30 min to allow excess compound to diffuse from cells. Then, cells were washed twice with PBS and incubated for 24h in complete medium containing or not 10µM Etoposide. Labelling of total CENP-A was then monitored by immunofluorescence as described below.

Immunofluorescence

Cells were grown on glass coverslips and treated as described in the drug treatment section followed by fixation with 4% paraformaldehyde in PBS (Electron Microscopy Sciences). After permeabilisation with 0.1% Triton X-100 in PBS, samples were blocked in 2% donkey serum (Jackson Immunoresearch) in PBS followed by incubation with primary antibodies (Table S3) in blocking buffer overnight at 4°C. After incubation with secondary antibodies conjugated to Alexa-Fluor 488 or 594 (Jackson ImmunoResearch), coverslips were mounted in Vectashield medium containing 2µg/ml DAPI (Vector Laboratories).

DNA & RNA FISH

Cells were directly grown on Superfrost Plus microscope slides (Menzel–Glaser, Braunschweig, Germany) then fixed and permeabilized as described above. For DNA-FISH, a denaturation step was added by incubating slides for 5 min in denaturation buffer (70%

formamide, 2X SCC) at 75°C. Slides for both DNA and RNA-FISH were dehydrated in successive ethanol baths (70, 90, 100%, 3 min each). Minor or major satellite oligonucleotide probes (Table S4) were labelled in 3' with fluorescent Cy3-dUTP or Alexa488-dUTP using Terminal Transferase (New England Biolabs) according to the manufacturer's recommendations. Slides were hybridized overnight at 37°C with 100 ng of labelled probe, 10 µg of yeast tRNA (Invitrogen) in Hybrisol VII buffer (MP Biomedicals). Slides were washed three times for 10 min in 2X SSC at 42°C and mounted in Vectashield medium with 2µg/ml DAPI (Vector Laboratories).

Of note, as permabilization of cells prior to fixation results in perturbations to nuclear architecture, in particular that of centromeric regions, control RNAse treatments for RNA-FISH experiments were not performed. Instead, we carefully verified that RNA-FISH signals for centromeric transcripts were undetectable prior to drug treatments. Hence, the increased signals that we detected matched PCR data and it was unlikely that they resulted from hybridization to DNA repeats. Likewise, RNA-FISH signals that spread throughout the nucleus cannot result from hybridization to centromeric DNA repeats.

Microscopy

Image acquisition was performed at room temperature on a fluorescence microscope (Axioplan 2; Zeiss) with a Plan-Neofluar 100X/1.3 NA oil immersion objective (Zeiss) using a digital cooled camera (CoolSNAP fx; Photometrics) and METAMORPH 7.04 software (Roper Scientific, Trenton, NJ). Images presented correspond to one focal plane. Analysis was performed by scoring at least 150 cells in each experiment.

siRNA and plasmid transfection

siRNA purchased from Sigma or Eurofins MWG Operon (Table S5) were transfected into cells using Interferin (Polyplus transfection) following manufacturer's instructions. Briefly, two rounds of transfection with synthetic siRNAs at a final concentration of 20 nM were performed: a first round on NIH/3T3 cells in suspension and the second when cells were allowed to adhere to the plate. Cells were collected 72 hr after the first transfection. Etoposide treatment was performed 48h post-transfection when this applies.

RNA extraction and RT-qPCR analysis

Total RNA from cell lines was isolated using TRI Reagent® (Sigma) according to manufacturer's instructions. Contaminant genomic DNA was eliminated with TURBO DNA-free kit (Ambion). Reverse transcription was carried out using 500ng DNA-free RNA and 50µM random hexamers (Roche), 20U of RNase inhibitor (New England Biolabs) and 20U of RevertAid Reverse Transcriptase (Fermentas). Complementary DNA reactions were used as templates for PCR reactions. Real-time PCR was performed using the LightCycler® DNA Master SYBR Green I mix (Roche) supplemented with 0.2µM specific primer pairs (Table S6). Real-time quantitative PCR were run on a light cycler rapid thermal system (LightCycler®480 2.0 Real time PCR system, Roche) with 20 sec of denaturation at 95°C, 20 sec of annealing at 60°C and 20 sec of extension at 72°C for all primers, and analyzed by the comparative CT(ΔΔCT) method using U6 RNA as an invariant RNA. As a control, complementary DNA reactions in which the reverse transcriptase was omitted were used to assess the absence of DNA contaminants in our templates. Primer efficiency was established by plotting a standard curve of Ct values from serial dilutions of cDNA and these were comparable for all DNA repeats. Therefore, the ΔΔCT was applied to compare variations in the levels of a given type of repeat transcripts without correcting for primer efficiency.

Nuclear extracts and Western blot

Cells were washed and scrapped in PBS. After centrifugation, cell pellets were lysed in RIPA buffer (300 mM NaCl, 1% NP-40, 0.5% Na-deoxycholate, 0.1% SDS, 50 mM Tris pH 8.0, complete protease inhibitor cocktail) for total extracts. For nuclear extracts, cells were resuspended in buffer H (10 mM Tris pH 7.9, 10 mM KCl, 1.5 mM MgCl2, 0.5% NP40, complete protease inhibitor cocktail) 20 min on ice to lyse cellular membranes. Nuclei were pelleted by centrifugation and lysed in RIPA buffer (300 mM NaCl, 1% NP-40, 0.5% Na-deoxycholate, 0.1% SDS, 50 mM Tris pH 8.0, complete protease inhibitor cocktail).

For Western blot analysis, proteins were resolved by SDS–PAGE, transferred onto PVDF membrane (Thermo Fischer) and probed using the appropriate primary (Table S3) and secondary antibodies coupled to horse-radish peroxidase (Jackson Immunoresearch). Protein detection was performed with ECL reagents (Pierce).

Chromatin Immunoprecipitation

Cells were directly cross-linked with 1% formaldehyde for 10min at room temperature (RT) and the reaction was quenched by adding glycine at a final concentration of 0.125M for 5min at RT. Fixed cells were washed and harvested with PBS. Cell membranes were lysed in cell lysis buffer (5mM Pipes pH8.0, 85mM KCl, 0.5% NP40, Complete protease inhibitor cocktail (Roche)) for 20min, 4°C. Nuclei were pelleted by centrifugation and lysed in nuclei lysis buffer (50mM Tris HCl pH8.1, 150mM NaCl, 10mM EDTA, 1% SDS, Complete protease inhibitor cocktail (Roche)) for 20min, 4°C. Chromatin was subjected to sonication with Bioruptor Power-up (Diagenode) yielding genomic DNA fragments with a bulk size of 150-300 bp. Supernatant was diluted 10 times in IP dilution buffer (16.7 mM Tris pH8, 167 mM NaCl, 1.2 mM EDTA, 1.1% Triton X-100, 0.01% SDS, complete protease inhibitor cocktail (Roche)). IPs were performed overnight at 4°C using 20µg of chromatin and 2µg of specific antibodies against indicated proteins (Table S3). Immune complexes were recovered by adding pre-blocked protein G magnetic beads (Dynabeads, Life Technologies) and incubated for 2h at room temperature. Beads were washed once with Low salt buffer (0.1% SDS, 1% Triton, 2 mM EDTA, 20 mM Tris pH 8, 150 mM NaCl), once with High salt buffer (0.1% SDS, 1% Triton, 2 mM EDTA, 20 mM Tris pH 8; 500 mM NaCl), once with LiCl wash buffer (10 mM Tris pH 8.0, 1% Na-deoxycholate, 1% NP-40, 250 mM LiCl, 1 mM EDTA) and twice with TE (10mM Tris pH 8.0, 1mM EDTA pH 8.0, 50mM NaCl). Beads were eluted in Elution buffer (50mM Tris pH 8.0, 10mM EDTA pH 8.0, 1% SDS) at 65°C for 45min and cross-link was reversed O/N at 65°C after addition of NaCl to 0.2M final concentration. The eluted material was digested with 40µg of Proteinase K (Invitrogen) at 65°c for 2h then phenol/chloroform-extracted and ethanol-precipitated. DNA was resuspended in water and q-PCR performed using LightCycler® DNA Master SYBR Green I mix (Roche) and analyzed on a light cycler rapid thermal system (LightCycler®480 2.0 Real time PCR system, Roche). ChIP-qPCR results were normalized on input signal (% input) then normalized against the untreated condition. Sequences of primers are listed in Table S6. As a negative control, an additional mock ChIP using IgG was systematically added to each experiment to verify check that no more than 0.01% of the input was precipitated whatever the genomic region tested, including satellite repeats.

Statistical analysis

For scoring CENP-A subnuclear localization, centromere architecture or transcription, cells were classified into categories indicated in each figure. A total of 300 to 400 cells from 2 to 3 experiments were analyzed, the proportion of cells assigned to each category was calculated, and category distributions were compared through a Chi-square test.

For qPCR analysis, quantitative data are presented as means ± SEM. Values were compared with the unpaired Student's t test, with a P value <0.05 being considered statistically significant. Representative data are the result of at least three independent experiments performed on at least three independent RNA extractions or chromatin preparations.

LEGENDS TO SUPPLEMENTAL FIGURES

Supplemental Figure S1. Characterization of cells subjected to genotoxic stress.

(A) Immunoblots of p53 and γH2AX proteins from nuclear extracts of NIH/3T3 cells treated with the indicated drugs in conditions commonly used: Etoposide (ETOP; 10µM, 4 hr), Zeocin (ZEO; 200µg/ml, 4 hr), Hydroxyurea (HU; 1mM, 18 hr), Mitomycin C (MMC; 10µg/ml, 4 hr treatment plus 24 hr recovery), UVC rays (20J, 24 hr after exposure) and Nocodazole (NOC; 10µM, 16 hr). Ponceau red staining served as a loading control. (B) FACS analysis of NIH/3T3 cells repartition in the cell-cycle after treatment with the indicated drugs and at the indicated time points.

Supplemental Figure S2. CENP-A is specifically delocalized in response to various genotoxic stress.

(A) Immunofluorescence analysis of CENP-A delocalization in response to different DNA damaging agents in conditions indicated in S1A. Percentage of counted cells that displayed CENP-A delocalization are indicated for each condition (n>300 cells). (B-C) Immunofluorescence analysis of (B) CENP-B and (C) CENP-C after a 24 hr ETOP treatment. (D) Immunoblots of p53, phosphorylated H2A.X (γH2AX) and cleaved Caspase-3 from total extracts of untreated NIH/3T3 cells (CTRL) or treated with ETOP at the indicated time points or following a 24 hr recovery period (4+24H). Ponceau red staining served as a loading control.

Supplemental Figure S3. CENP-A delocalization correlates with persistence of stress signalling.

(A) Percentage of counted cells that displayed CENP-A delocalization as in S2A are shown for the indicated drugs, after a 24 hr treatment, or after a 4 hr treatment followed by a 24 hr recovery period. (n>300 cells). P-values (unpaired t-test) comparing control (CTRL) and drug treatments: *** $p<0.001$; ** $p<0.01$. (B) Immunoblots of γH2AX from total extracts of untreated NIH/3T3 cells (CTRL) or treated with the indicated drug at the indicated time points or following a 24 hr recovery period (4+24). Ponceau red staining served as a loading control. (C) FACS analysis of NIH/3T3 cells repartition in the cell-cycle following a 24 hr ETOP

treatment in the absence of or concomitant with inhibition of DDR signalling using inhibitors of ATM (ATMi) or ATR (ATRi).

Figure S4. Transcriptional analysis of centromeric and other DNA repeats in stress conditions.

(A) The histogram represents the results of RT-qPCR analysis of minor satellite repeats expression in NIH/3T3 cells treated with the indicated drugs in conditions used in S1A. Additional conditions: heat shock (HS; 1hr at 42°C followed by 1h at 37°C), and Ethanol (EtOH; 1%, 8 hr). Below is a western blot analysis of the induction of the DNA damage repair pathway in nuclear extracts from NIH/3T3 following the various drug treatments indicated in the upper graph, characterized by p53 and $p21^{CIP}$ stabilization and phosphorylation of H2AX (γH2AX) in stress conditions generating DNA strand breaks. Ponceau red served as a loading control (B) qRT-PCR analysis of expression of murine DNA repeats during the kinetics of ETOP treatment. Ribosomal DNA (rDNA); interspersed repeats: long (LINE) and short (SINE) interspersed nuclear elements; transposable elements: intracisternal A-particle (IAP); telomeric repeats from juxtacentromeric (TeloCen) or long arm of chromosome 11 (11q) positions. Histograms represent relative fold change values normalized to U6 RNA levels and compared to untreated cells (CTRL). Error bars represent S.E.M of at least three independent experiments. P-values (unpaired t-test) comparing control (CTRL) and drug treatments: *** p<0.001; ** p<0.01; *p<0.05.

Supplemental Figure S5. Transcription is required but centromeric transcripts are not sufficient to promote CENP-A delocalization.

(A) qRT-PCR analysis of minor satellites expression and (B) Immunofluorescence analysis of CENP-A delocalization in NIH/3T3 cells treated for 24 hr with Etoposide (ETOP, 10µM) in presence or not of the RNA polymerase inhibitor Actinomycin D (Act D; 5µg/ml). Error bars represent S.E.M of at least three independent experiments. (C) qRT-PCR analysis of minor satellites ectopic expression 24 hr after transfection of NIH/3T3 cells with a plasmid containing four minor satellite repeats under the control of the CMV promoter (MIN SAT) or a control plasmid transcribing a fragment of irrelevant RNA of the same size (CTRL). (D) Immunofluorescence analysis of CENP-A delocalization 24 hr after transfection of NIH/3T3

cells with the minor satellite (MIN SAT) or control (CTRL) expression vectors. P-values (unpaired t-test) comparing control (CTRL) and drug treatments: ** $p<0.01$; *$p<0.05$.

Supplemental Figure S6. The DDR effector p53, but not ATM/ATR, are required for transcriptional activation of minor satellite repeats in response to genotoxic stress.

(A) qRT-PCR analysis of minor satellites expression in NIH/3T3 cells with Etoposide (10µM) for 24 hr in presence or not of ATM (ATMi) or ATR (ATRi) inhibitors. P-values (unpaired t-test) comparing controls (CTRL) and drug treatments: ** $p<0.01$. (B) RT-qPCR analysis of $p21^{CIP}$ and minor satellite transcripts after treatment with Etoposide at the indicated concentration for 4 hr in $p53^{+/+}$ (NIH/3T3) or $p53^{-/-}$ fibroblasts, and immunoblots of p53 and γH2AX proteins from nuclear extracts of cells treated as above. Ponceau red staining served as a loading control. P-values (unpaired t-test) comparing controls (CTRL) and drug treatments: *** $p<0.001$; ** $p<0.01$. (C) Analysis of $p21^{CIP}$ and minor satellite transcripts after treatment of NIH/3T3 cells with Etoposide (E), Nutlin-3 (N3) or both (E+N3), for 4 hr, using RT-qPCR. Immunoblot of p53 and γH2AX from nuclear extracts of cells treated as above. Ponceau red staining served as a loading control. P-values (unpaired t-test) comparing controls (CTRL) and drug treatments: *** $p<0.001$; ** $p<0.01$. (D) FACS analysis of $p53^{-/-}$ cells repartition in the cell-cycle after ETOP treatment at the indicated time points compared to untreated cells (CTRL). Error bars represent S.E.M of at least three independent experiments.

Supplemental Figure S7. CENP-A delocalization is dependent on chromatin remodelers.

qRT-PCR analysis of siRNA-mediated knockdown efficiency for each chromatin remodelling factors in untreated (CTRL) and cells treated with etoposide for 24 hr (ETOP). Values represented are relative fold change compared to untreated (CTRL) control siRNA (siCTRL) and normalized against U6 RNA levels. Error bars represent S.E.M of at least three independent experiments.

Supplemental Figure S8. Increased transcription of minor satellite transcripts in stressed or senescent MEFS

(A) qRT-PCR analysis of minor satellites expression and the senescence marker $p16^{Ink4}$ in primary MEFs treated for the indicated time with Etoposide (ETOP, 2µM). Values represented are relative fold change compared to untreated (CTRL) cells and normalized against U6 RNA levels. Error bars represent S.E.M of at least three independent experiments. P-values (unpaired t-test) comparing controls (CTRL) and drug treatments: * $p<0.05$ (unpaired t-test). (B) Percentage of primary MEFs treated as in A that exhibit CENP-A delocalization. P-values (Chi-square test) comparing controls (CTRL) and drug treatments: *** $p<0.001$. (C) Senescence-associated β-Galactosidase staining in primary mouse embryonic fibroblasts (MEFs) at passage 2 and 6. Senescent cells are stained in blue. (D) Western blot analysis of markers of senescence: p53, $p21^{CIP}$ and $p16^{INK4}$ in primary MEFs at passage 2 (P2) and 6 (p6). Levels of γ-tubulin served as a loading control. (E) Western blot analysis of markers of senescence p53, $p21^{CIP}$ and $p16^{INK4}$ after treatment of primary MEFs at p2 with 10µM Nutlin-3 for the indicated time points. Levels of α-tubulin served as a loading control.

Supplemental Figure S9. Transcriptional, structural and epigenetic disorganization of centromeric repeats in senescent MEFs

(A) Analysis of minor satellites transcription in senescent MEF cells at passage 6 (p6), using RT-qPCR. Data are normalized to U6 RNA levels and compared to primary MEFs at passage 2 (p2). Error bars represent S.E.M of at least three independent experiments. P-values (unpaired t-test) comparing MEFp2 and p6: * $p<0.05$ (unpaired t-test). (B) Analysis of minor satellites transcription in senescent MEF after treatment with Nutlin-3 (10µM, 72hr), using RT-qPCR. Data are normalized to U6 RNA levels and compared to untreated primary MEFs at passage 2 (p2). Error bars represent S.E.M of at least three independent experiments. P-values (unpaired t-test) comparing untreated and treated MEFs: * $p<0.05$ (unpaired t-test). (C) Analysis of CENP-A localization in senescent MEFs at passage 6 compared to primary cells at passage 2 using anti-CENP-A antibodies and FITC-conjugated secondary antibodies. Insets are magnified an additional 3 fold. Scale bar, 10µm. The percentage of cells showing a fragmented pattern (as in C; middle panel) or both g-fragmented pattern and a CENP-A nucleolar staining (as in C; lower panel) are represented on the histogram (n>200 cells). P-

values (Chi-square test) comparing MEFp2 and p6: *** $p<0.001$. (D) Disorganization of minor and major satellites nuclear domains in senescent MEFs (p6) compared to primary MEFs (p2) as revealed by DNA-FISH using specific directly labelled probes. Scale bar, 10µm. The percentage of cells showing stretched signals for minor and major satellite repeats are represented on the histogram (n>200 cells). P-values (Chi-square test) comparing MEFp2 and p6: *** $p<0.001$.

SUPPLEMENTAL TABLES

Supplemental Table S1: List of drugs used in the study, the type of DNA lesions that they promote and their impact on cell-cycle

DRUG	DRUG abbreviation	TYPE OF DNA LESION	PHASE OF EXPECTED CELL CYCLE ARREST
**Etoposide**	**ETOP**	Double Strand Breaks	G2/M
**Zeocin**	**ZEO**	Single Strand Breaks<br>Double Strand Breaks	G2/M
**Hydroxyurea**	**HU**	Replication fork stalling	G1/S
**Mitomycin C**	**MMC**	DNA crosslink	S, G2/M
**Ultraviolet C**	**UVC**	Pyrimidine dimers	G1/S
**Nocodazole**	**NOC**	Inhibition of spindle microtubule formation	G2/M

Supplemental Table S2: List of chromatin remodelers and histone chaperones tested in loss of function experiments

NAME	ASSOCIATED COMPLEX	(PERI)CENTROMERIC LOCALIZATION	REFERENCES
**CHD3**	NuRD (Mi-2)	Pericentromeres	Helbling Chadwick et al., 2009<br>Sims and Wade, 2011
**CHD4**			
**BRG1**	SWI/SNF	Pericentromeres, centromeres	Nielsen et al., 2002<br>Gkikopoulos et al., 2011<br>Bourgo et al., 2009
**INO80**	INO80	Pericentromeres, centromeres	Ogiwara et al., 2007<br>Chambers et al., 2012
**ATRX**	DAXX/ATRX	Pericentromeres, centromeres	Pluta et al., 1998<br>McDowell et al., 1999<br>Morozov et al., 2012<br>Lacoste et al., 2014
**DAXX**			
**HELLS/LSH**		Pericentromeres, centromeres	Dennis et al., 2001<br>Yan et al., 2003
**SSRP1**	FACT	Pericentromeres, centromeres	Foltz et al., 2006<br>Ishov et al., 2004<br>Lejeune et al., 2007<br>Okada et al., 2009

Supplemental Table S3. Primary Antibodies used in immunofluoscence and western blots.

TARGET	HOST	SUPPLIER/REFERENCE	APPLICATION
**B23 (Nucleophosmin)**	mouse	Santa Cruz Biotechnology (sc56622)	IF
**CENP-A**	rabbit	Abcam (ab33565)	ChIP, WB
**CENP-A**	rabbit	Cell Signaling Technology (2048)	IF
**CENP-B**	rabbit	Santa Cruz Biotechnology (sc22788)	IF
**CENP-C**	rabbit	Gift from P. Kalitsis	IF
**γH2AX (pS139)**	rabbit	Epitomics (2212-1)	WB
**Histone H2A**	rabbit	Cell Signaling Technology (12349)	ChIP
**Histone H3**	rabbit	Abcam (ab1791)	ChIP
**Histone H4**	rabbit	Abcam (ab10158)	ChIP
**p53**	rabbit	Santa Cruz Biotechnology (sc-6243)	WB
**p21**	mouse	Santa Cruz Biotechnology (sc-6246)	WB
**p16**	rabbit	Santa Cruz Biotechnology (sc-1207)	WB
**γ-tubulin**	mouse	Sigma (T6557)	WB
**SSRP1**	mouse	Biolegend (609701)	WB
**Cleaved Caspase-3**	rabbit	Cell Signaling Technology (9661)	WB, IF

Supplemental Table S4: List of oligonucleotide probes used in DNA or RNA-FISH assays.

NAME	SEQUENCE (5' to 3')	ASSAY
Minor satellites probe	ATGTTTCACATTGTAACTCATTGATATACACTGTTCTACAA ATCCCGTTTCCAACGAATGTGTTTT	RNA-FISH, DNA-FISH
Major satellites probe	AAATATGGCGAGGAAAACTGAAAAAGGTGGAAAATTTA GAAATGTCCACTGTAGGACGTG	DNA-FISH

Supplemental Table S5. Sequences of siRNA used in loss of function experiments.

siRNA	Target sequence (5' -> 3')
**siATRX**	GAUGCUAGAUCAUCAGUAA
**siBRG1**	CAAACUGGGCGUAUGAAUU
**siCHD3**	CCGCCGCAGGCGCCAAUUA
**siCHD4**	GUCUAAGUUCUUUCGAGUU
**siCtrl**	GCCGGUAUGCCGGUUAAGU
**siDAXX**	GUAACUCCGGUAGUAGGAA
**siINO80**	GAAACUUAUUUCUUACCAA
**siLSH**	GAGAAGUUGAUAAUCCAUA
**siSSRP1**	CAGUGUACCACAGGCAAGA

Supplemental Table S6. Sequences of primers used for RT-qPCR analysis.

(Primers specific for repeat families: # primers from Bouzinba-Segard et al. 2006; * primers from Martens et al. 2005; § primers from Deng et al. 2012)

PRIMER NAME	SEQUENCE 5'-> 3'	APPLICATION
Minor satellites F #	GAACATATTAGATGAGTGAGTTAC	RTqPCR, ChIP
Minor satellites R #	GTTCTACAAATCCCGTTTCCAAC	
Major satellites F *	GACGACTTGAAAAATGACGAAATC	RTqPCR, ChIP
Major satellites R *	CATATTCCAGGTCCTTCAGTGTGC	
rDNA F (intergenic spacer) *	CCTGTGAATTCTCTGAACTC	RTqPCR, ChIP
rDNA R (intergenic spacer) *	CCTAAACTGCTGACAGGGTG	
LINE-1 family F (ORF2) *	TTTGGGACACAATGAAAGCA	RTqPCR
LINE-1 family R (ORF2) *	CTGCCGTCTACTCCTCTTGG	
SINE B1 family F *	GTGGCGCACGCCTTTAATC	RTqPCR
SINE B1 family R *	GACAGGGTTTCTCTGTGTAG	
SINE B2 family F *	GAGATGGCTCAGTGGTTAAG	RTqPCR
SINE B2 family R*	CTGTCTTCAGACACTCCAG	
IAP family F (GAG) *	AGCAGGTGAAGCCACTG	RTqPCR
IAP family R (GAG) *	CTTGCCACACTTAGAGC	
TERRA TeloCen F §	CCAAAGTTTCTGCAAGGCAAA	RTqPCR
TERRA TeloCen R §	CCCAATCTGTTGGTGGTCTTTT	
TERRA 11q F §	TGCCATTGGAACACAGCAA	RTqPCR
TERRA 11q R §	CGTCTGCTGAGGTCCACAGA	
CHD3 F	AGCCCGCCTTCAGATGTC	RTqPCR
CHD3 R	GGAGGTGTGGCGTAAGGAC	
CHD4 F	GGCCATCTTGGGTTCTATTG	RTqPCR

CHD4 R	AACAGCTTGTGCTGGAGTGA	
BRG1 F	GACCAGCCCCTCCAAATTAC	RTqPCR
BRG1 R	CTTCTGTGGGGTGCTTGTG	
INO80 F	TTTCTTACCAATAGCCGAAAGC	RTqPCR
INO80 R	TCGAGGCAGAGTCTCCTTACA	
ATRX F	AGCCGTGACTCAGATGGAAT	RTqPCR
ATRX R	CGCAGGATGCATTTCTTACA	
LSH F	ACACGAGCTGGTGGTTTAGG	RTqPCR
LSH R	GCCTGGAGATCAGACTGAGG	
DAXX F	GTGGATGGAGGGAGCAGTAA	RTqPCR
DAXX R	AGTTTGTGGAGGAACGGAAC	
SSRP1 F	TGGGCTTAAACTGCTCACAA	RTqPCR
SSRP1 R	TCCATTAGCTCAAGGCGATA	
U6 F	CTCGCTTCGGCAGCACA	RTqPCR
U6 R	AACGCTTCACGAATTTGCGT	

SUPPLEMENTAL REFERENCES

Bourgo, R.J. et al. SWI/SNF deficiency results in aberrant chromatin organization, mitotic failure, and diminished proliferative capacity. *Mol. Biol.* Cell **20**, 3192–3199 (2009).

Bouzinba-Segard, H., Guais, A., & Francastel, C. Accumulation of small murine minor satellite transcripts leads to impaired centromeric architecture and function. *Proc. Natl. Acad. Sci. U. S. A.* **103**, 8709–8714 (2006).

Chambers, A.L. et al. The INO80 chromatin remodeling complex prevents polyploidy and maintains normal chromatin structure at centromeres. *Genes Dev.* **26**, 2590–2603 (2012).

Deng, Z. et al. Formation of telomeric repeat-containing RNA (TERRA) foci in highly proliferating mouse cerebellar neuronal progenitors and medulloblastoma. *J Cell Sci.* 125, 4383-4394 (2012).

Dennis, K., Fan, T., Geiman, T., Yan, Q., and Muegge, K. Lsh, a member of the SNF2 family, is required for genome-wide methylation. *Genes Dev.* **15**, 2940–2944 (2001).

Foltz, D.R. et al. The human CENP-A centromeric nucleosome-associated complex. *Nat. Cell Biol.* **8**, 458–469 (2006).

Gkikopoulos, T. et al. The SWI/SNF complex acts to constrain distribution of the centromeric histone variant Cse4. *EMBO J.* **30**, 1919–1927 (2011).

Helbling Chadwick, L., Chadwick, B.P., Jaye, D.L., and Wade, P.A. The Mi-2/NuRD complex associates with pericentromeric heterochromatin during S phase in rapidly proliferating lymphoid cells. *Chromosoma* **118**, 445–457 (2009).

Ishov, A.M., Vladimirova, O.V., and Maul, G.G. Heterochromatin and ND10 are cell-cycle regulated and phosphorylation-dependent alternate nuclear sites of the transcription repressor Daxx and SWI/SNF protein ATRX. *J. Cell Sci.* **117**, 3807–3820 (2004).

Lacoste, N. et al. Mislocalization of the Centromeric Histone Variant CenH3/CENP-A in Human Cells Depends on the Chaperone DAXX. Mol. *Cell* **53**, 631–644 (2014).

Lejeune, E. et al. The Chromatin-Remodeling Factor FACT Contributes to Centromeric Heterochromatin Independently of RNAi. *Curr. Biol.* **17**, 1219–1224 (2007).

Martens, JH. et al. The profile of repeat-associated histone lysine methylation states in the mouse epigenome. *EMBO J.* **24**,800-812 (2005).

McDowell, T.L. et al. Localization of a putative transcriptional regulator (ATRX) at pericentromeric heterochromatin and the short arms of acrocentric chromosomes. Proc. Natl. Acad. Sci. *96*, 13983–13988 (1999).

Morozov, V.M., Gavrilova, E.V., Ogryzko, V.V., and Ishov, A.M. Dualistic function of Daxx at centromeric and pericentromeric heterochromatin in normal and stress conditions. *Nucleus* **3**, 276–285 (2012).

Nielsen, A.L. et al. Selective interaction between the chromatin-remodeling factor BRG1 and the heterochromatin-associated protein HP1α. *EMBO J.* **21**, 5797–5806 (2002).

Ogiwara, H., Enomoto, T., and Seki, M. The INO80 Chromatin Remodeling Complex Functions in Sister Chromatid Cohesion. *Cell Cycle* **6**, 1090–1095. (2007).

Okada, M., Okawa, K., Isobe, T., and Fukagawa, T. CENP-H–containing complex facilitates centromere deposition of CENP-A in cooperation with FACT and CHD1. *Mol. Biol. Cell* **20**, 3986–3995 (2009).

Pluta, A.F., Earnshaw, W.C., and Goldberg, I.G. Interphase-specific association of intrinsic centromere protein CENP-C with HDaxx, a death domain-binding protein implicated in Fas-mediated cell death. *J. Cell Sci.* **111**, 2029–2041 (1998).

Sims, J.K., and Wade, P.A. Mi-2/NuRD complex function is required for normal S phase progression and assembly of pericentric heterochromatin. *Mol. Biol. Cell* **22**, 3094–3102 (2011).

Yan, Q., Cho, E., Lockett, S., and Muegge, K. Association of Lsh, a Regulator of DNA Methylation, with Pericentromeric Heterochromatin Is Dependent on Intact Heterochromatin. *Mol. Cell. Biol.* **23**, 8416–8428 (2003).

Figure S1

Hedouin et al.

A

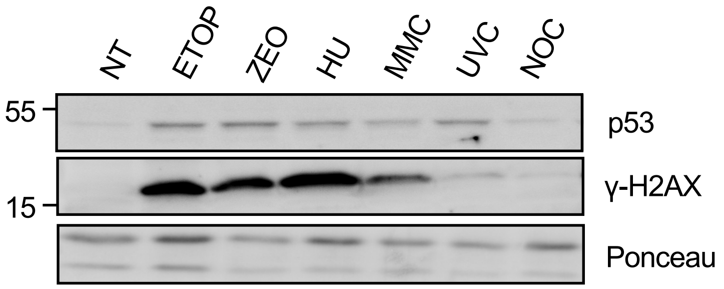

B

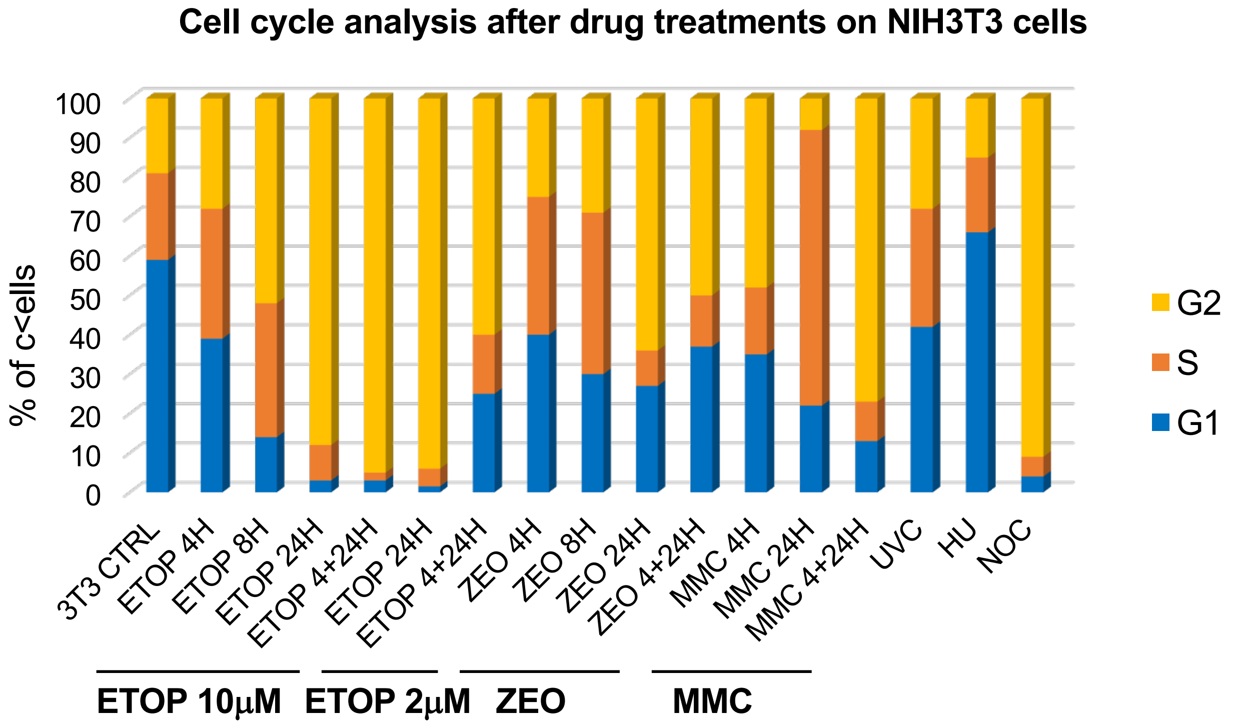

Figure S2

Hedouin et al.

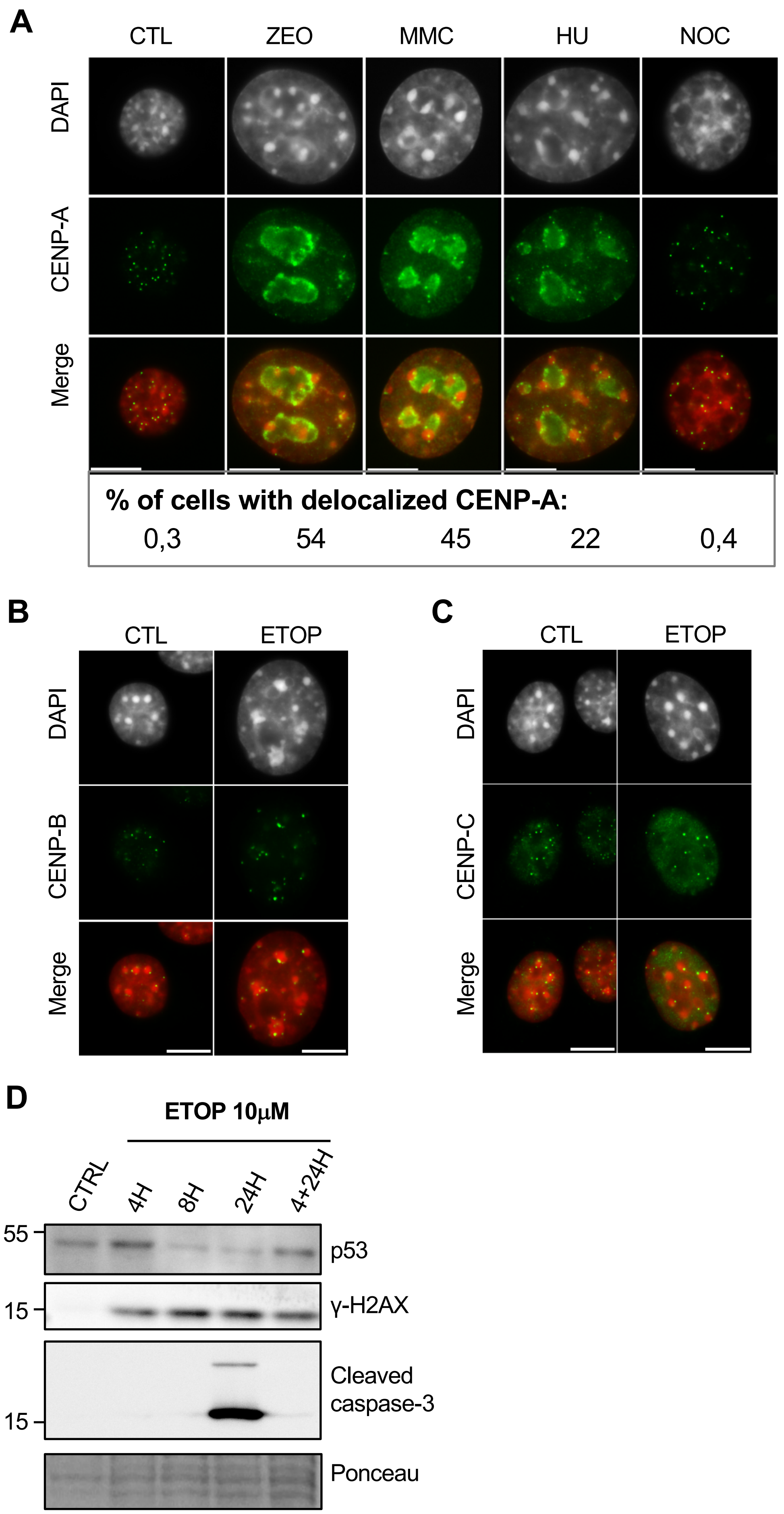

Figure S3 Hedouin et al.

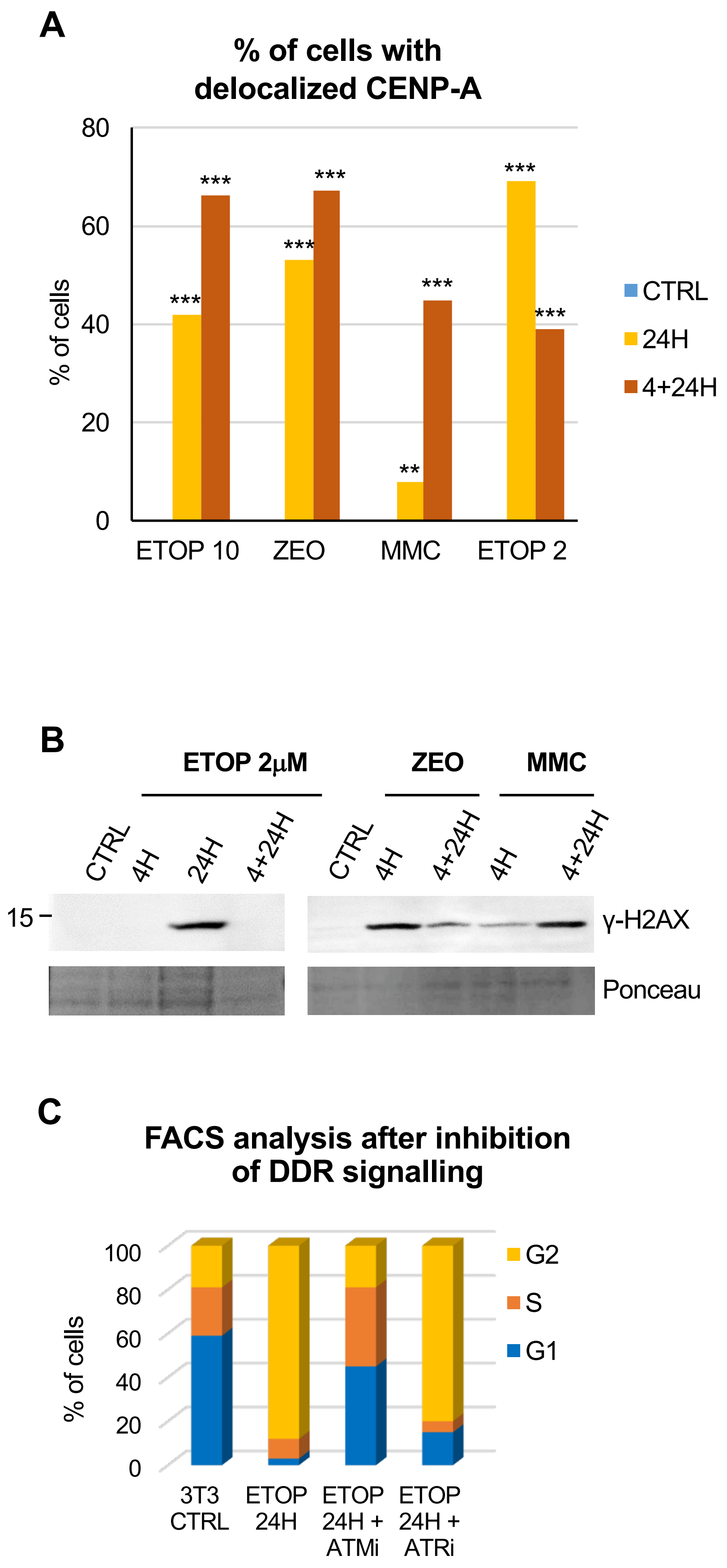

Figure S4

Hedouin et al.

A

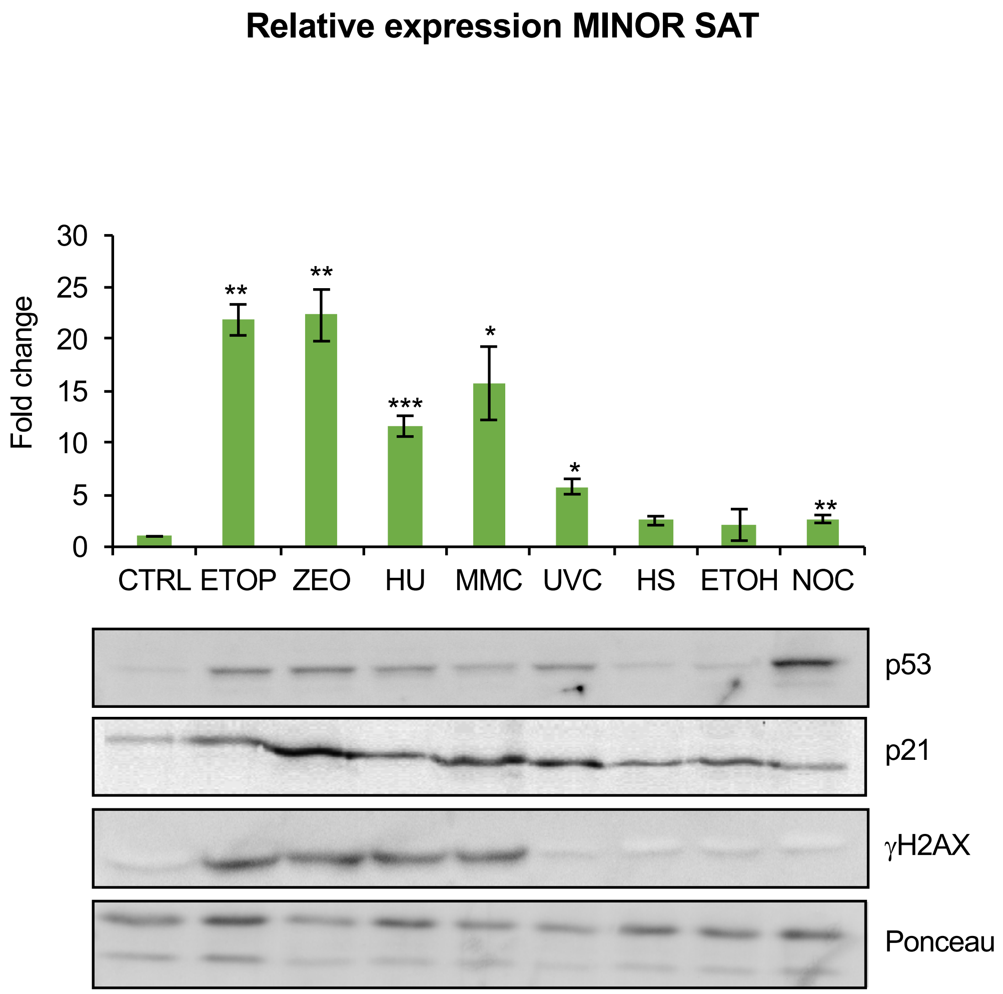

B

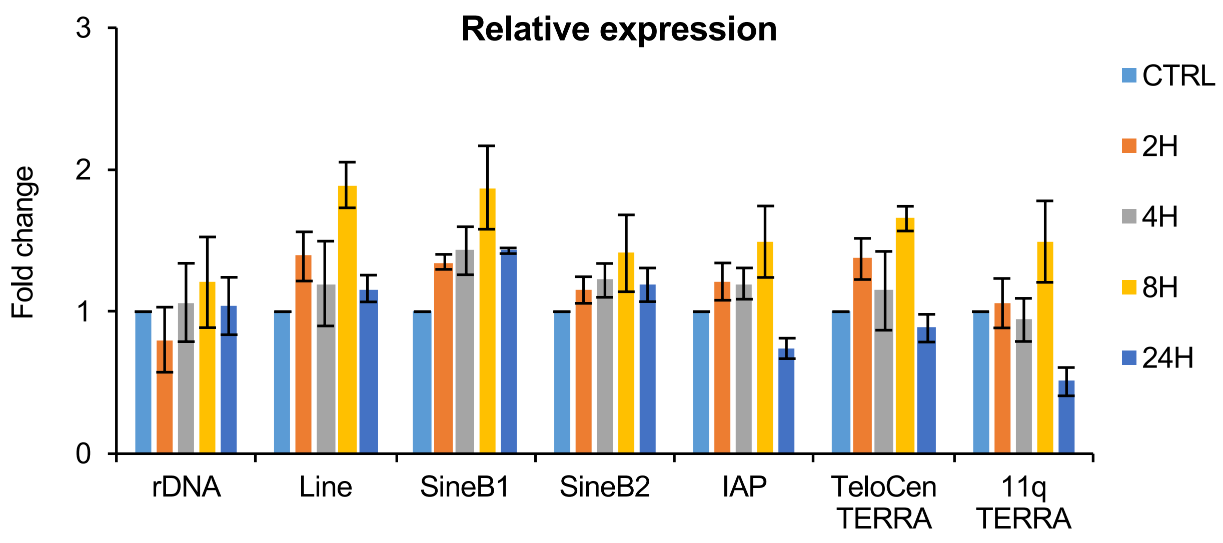

Figure S5

Hedouin et al.

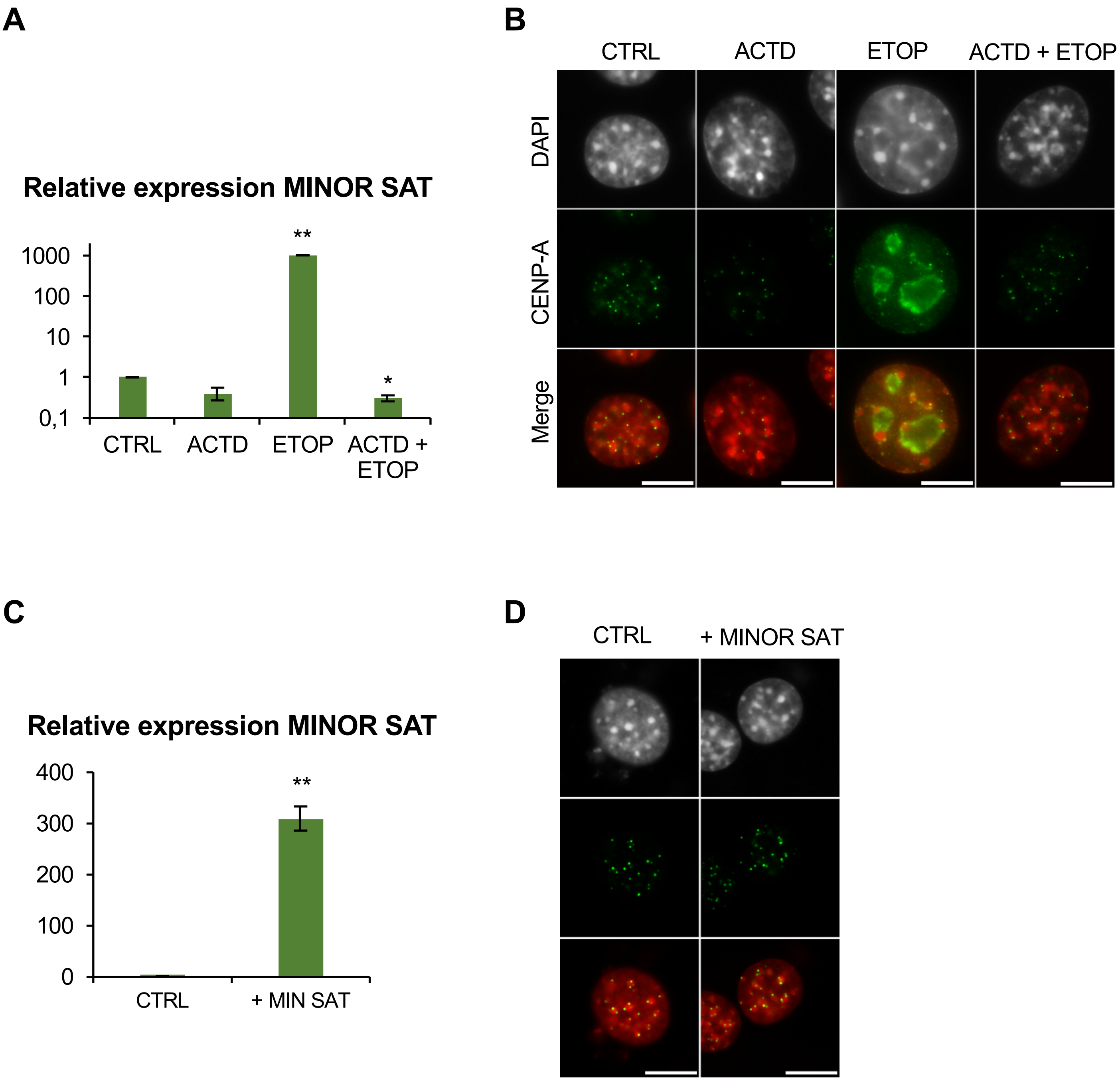

Figure S6

Hedouin et al.

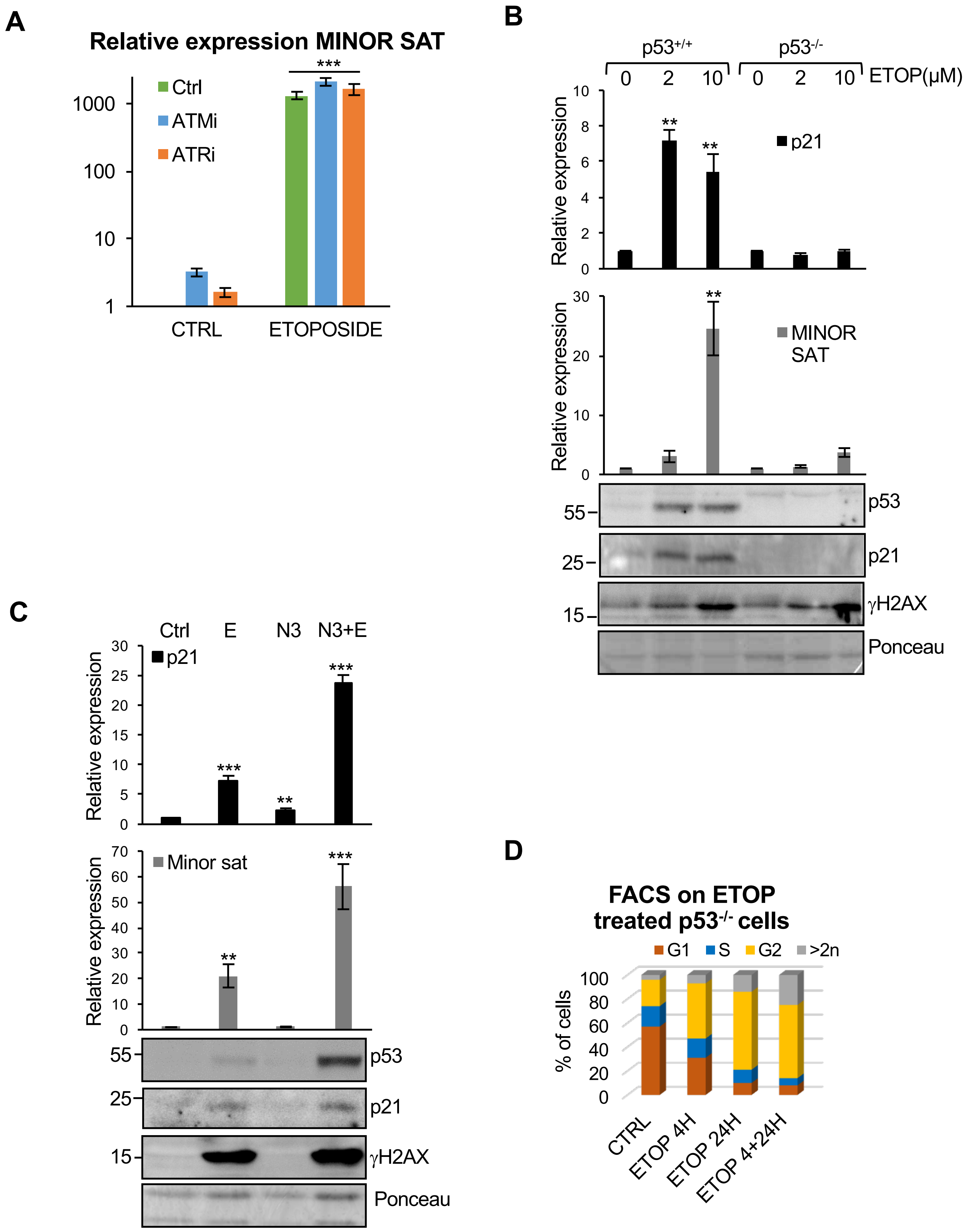

Figure S7

Hedouin et al.

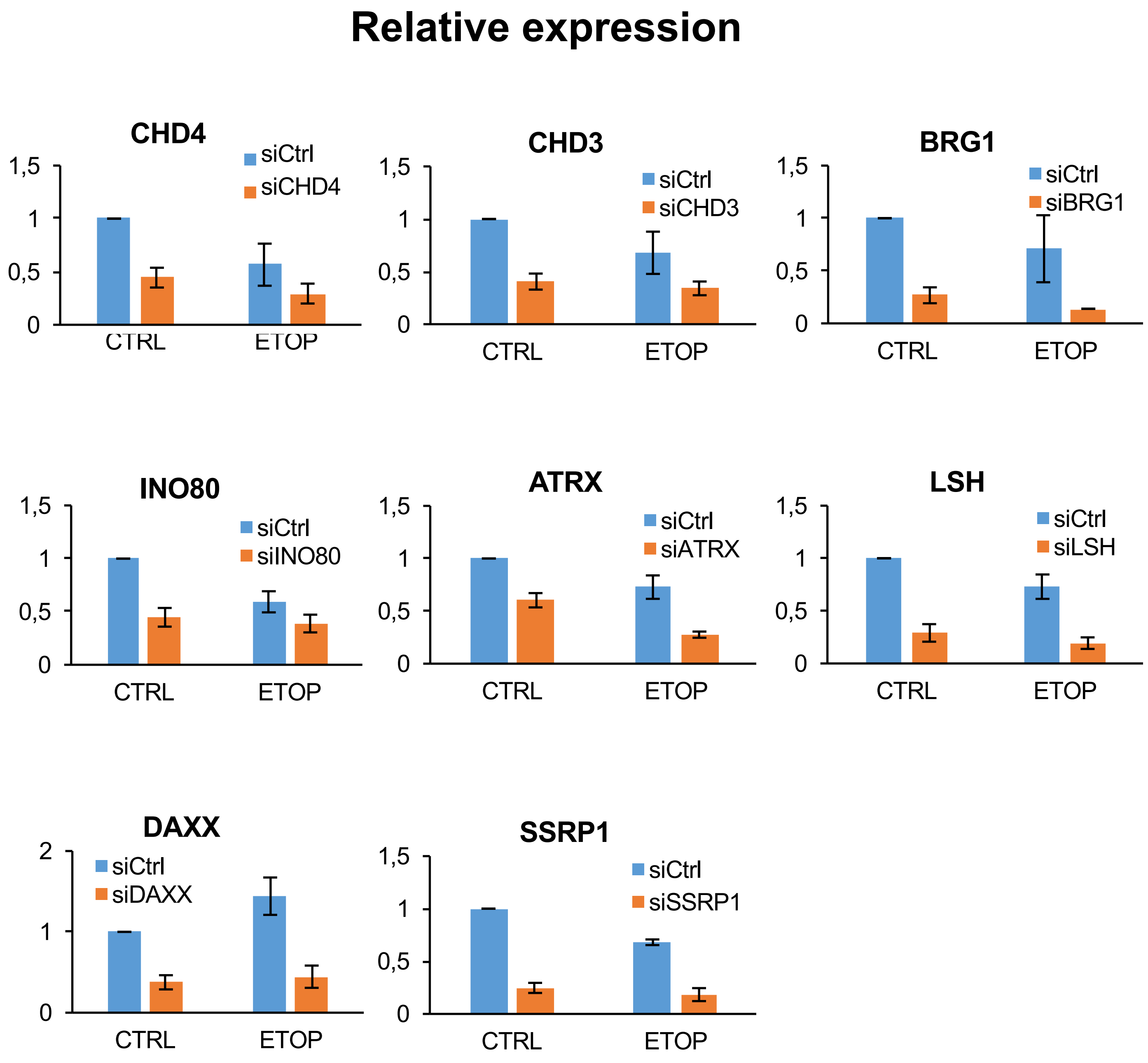

Figure S8

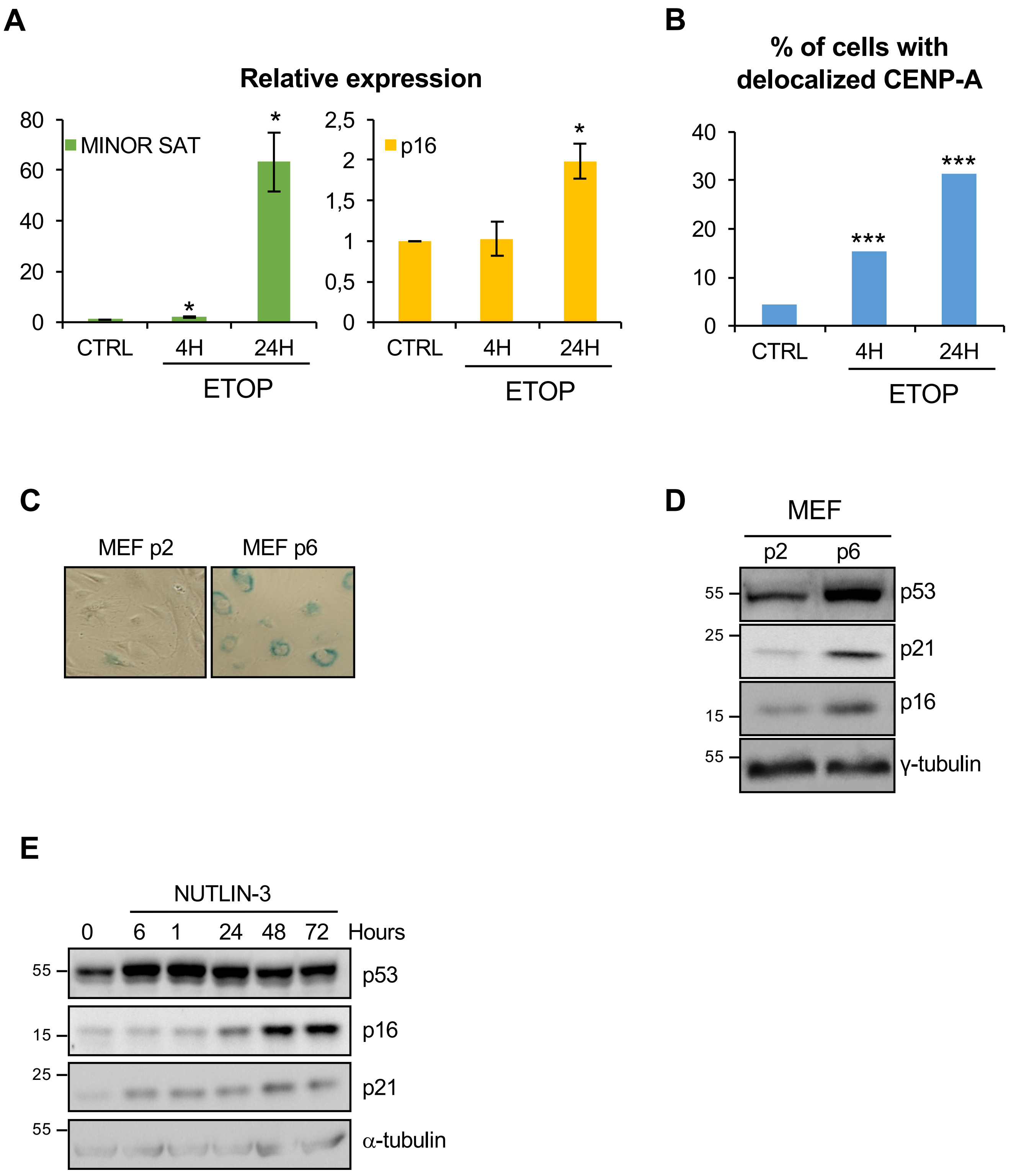
A
Relative expression
MINOR SAT
p16
80
60
40
20
0
2,5
2
1,5
1
0,5
0
*
CTRL
4H
24H
ETOP
B
% of cells with delocalized CENP-A
40
30
20
10
0
***
CTRL
4H
24H
ETOP
C
MEF p2
MEF p6
D
MEF
p2
p6
55
25
15
55
p53
p21
p16
γ-tubulin
E
NUTLIN-3
0
6
1
24
48
72
Hours
55
15
25
55
p53
p16
p21
α-tubulin

Figure S9

Hedouin et al.

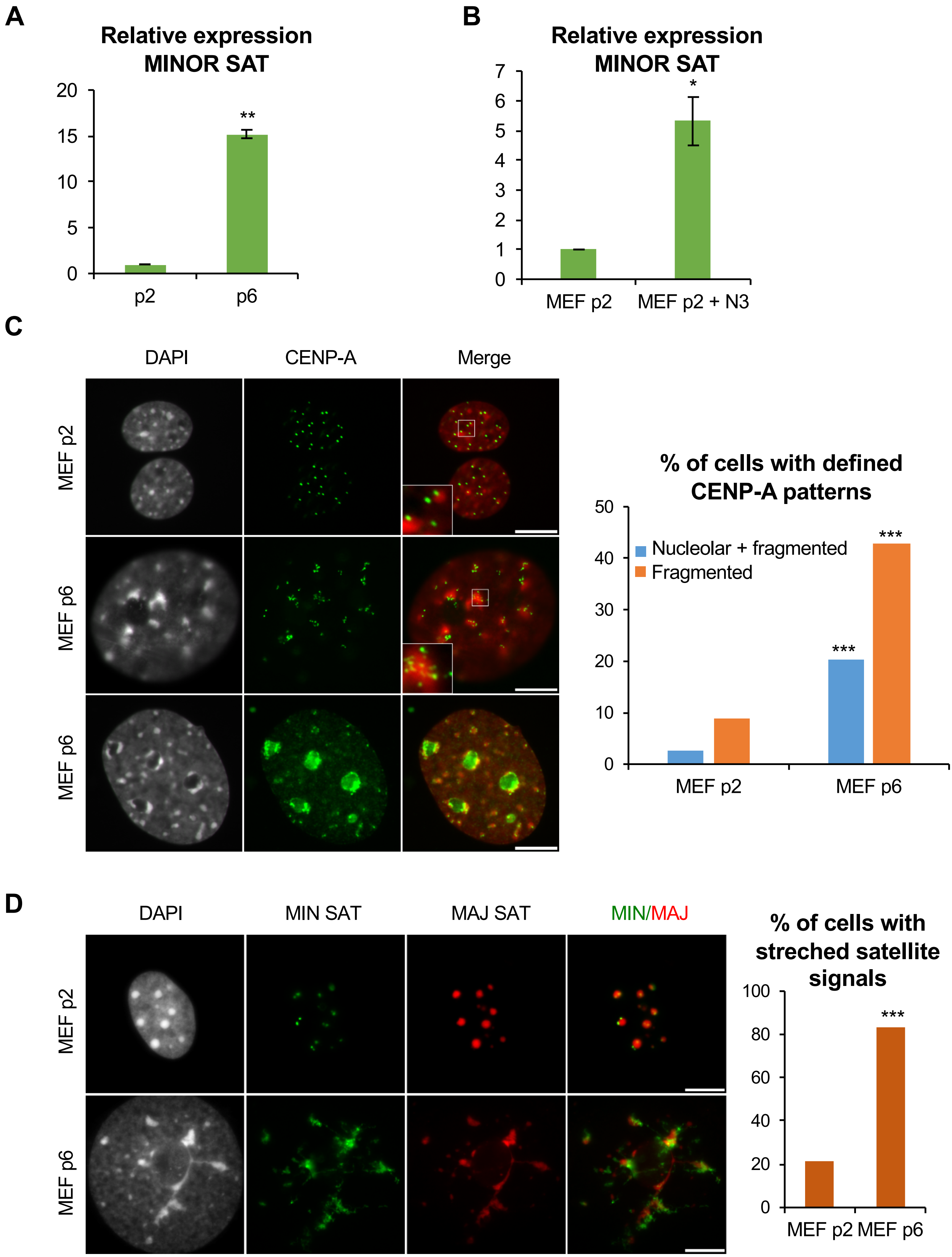

Supplement: Supplementary Information and Figures [file srep42520-s1.pdf]
